# Development and modeling of a novel type of photoreactors with exterior ultraviolet (UV) reflector for water treatment applications

**DOI:** 10.1038/s41598-023-34799-0

**Published:** 2023-05-11

**Authors:** Amirhossein Hassanpour, Alireza Jalali, Mehrdad Raisee, Mohammad Reza Naghavi

**Affiliations:** School of Mechanical Engineering, College of Engineering, University of Tehran, Tehran, Iran

**Keywords:** Engineering, Mathematics and computing

## Abstract

Ultraviolet (UV) water disinfection method has emerged as an alternative to chemical methods of disinfection. In typical UV photoreactors for water treatment, water flows in the space between the lamp’s sleeve and outer shell. The contact of water and sleeve causes fouling, which reduces the effectiveness of UV. To clean the photoreactor, the quartz sleeve must be replaced; this may lead to quartz or lamp breakage and mercury leakage into water during cleaning. In this study, a novel type of multi-lamp UV photoreactors is proposed, in which the UV lamps are placed out of the water channel and their UV irradiation is redirected into the channel using an outer cylindrical reflector. This allows for the installment of a self-cleaning mechanism for the water channel. A well-validated three-dimensional CFD model is utilized to model the performance of this photoreactor for microbial inactivation. The impacts of several geometrical and optical parameters are investigated on the inactivation of microorganisms. The results revealed that the difference in log reduction values (LRV) between fully specular and fully diffuse reflector ranges from 10 to 47% as the lamp-to-channel distance increases. For the volumetric flow rate of 25 GPM, the LRV of a photoreactor with fully diffuse reflector can be 46% higher than a fully specular one. In addition, the performance of the proposed photoreactor is compared against a classic L-shaped annular photoreactor. The results show that the new design can provide equal or better microbial performance compared to the classic photoreactor, but it removes many of their common issues such as quartz fouling, lamp overheating at low flow rates, and sleeve breakage during lamp replacement.

## Introduction

Water is widely recognized as the elixir of life since it is essential for people, industry, electricity production, and agriculture. Even though water covers 71% of the earth's surface, barely its 0.8% is accessible as surface and subsurface freshwater^1^. As a result, people in impoverished nations have long struggled to find clean, reliable drinking water supplies.

Surface water has to be treated before it can be used for drinking^2^. One of the essential water treatment procedures is disinfection, which guarantees water is free of hazardous microorganisms that cause waterborne illnesses^3,4^. Various procedures are utilized for disinfection, including chlorination, ozonation, and ultra-violet light (UV) disinfection. With the exception of the usage of UV, each of these procedures is chemical^5^. The most prevalent disinfection method is chlorination^6^. However, the creation of hazardous by-products (e.g., trihalomethanes) and the massive chlorine dosages often necessary for successful disinfection are the serious disadvantages of this chemical method^7^. Moreover, at the doses generally employed for water disinfection, certain microorganisms, such as *Cryptosporidium parvum*, are relatively resistant to free chlorine. The primary drawbacks of ozonation are the potential for producing bromates and aldehydes by-products and high energy consumption^8,9^.

UV disinfection prevents pathogens reproduction by penetrating into their genetic material. As a result, instead of being a chemical process, it is a physical one^10^. Not only is UV disinfection an efficient and secure approach to inactivate tenacious microorganisms, but it is also a chemical-free means of disinfecting water.

With significant improvements in computing power over the past two decades, computational fluid dynamics (CFD), a sophisticated modeling technique used in aerospace and mechanical engineering applications, has gained much interest in water treatment applications^11,12^. This technique has been used in several studies for water disinfection with UV^13–16^.There are three steps for the simulation of UV water disinfection process: simulation of flow field, UV radiation field, and pathogen inactivation process. The Lagrangian and Eulerian frameworks are the two standard techniques that may be used to model UV reactors using CFD. Microorganisms are regarded as a scattered phase in the Lagrangian method in which their inactivation is determined independently for each trajectory, and overall inactivation is provided as an ensemble average. On the other hand, in the Eulerian method, microorganisms are regarded as part of a continuous phase, whose concentration is obtained by the solution of a convection–diffusion equation within the computational domain^17^.

Flow hydrodynamics modeling is a significant area of research for the design of UV photoreactors. In a study investigating the flow field in UV reactors, three turbulence models were used by Sozzi and Taghipour^18^. The results revealed that the realizable *k*–*ε* model had the highest agreement with experimental data. Furthermore, their analysis of two annular reactors using both the Eulerian and Lagrangian methodologies indicated that, at high flow rates, the results of the two methods are very similar. However, there were some inconsistencies at lower flow rates^19^. Six turbulence models were used by Liu et al.^20^ to predict the flow field in a cross-flow UV reactor. They observed that the findings achieved in the wake area utilizing the standard *k*–*ω* and SST *k*–*ω* models were in excellent agreement with experiments.

To estimate the performance of UV reactors accurately, a precise assessment of the UV radiation field is essential. Liu et al.^21^ discovered that the discrete ordinates (DO) radiation model overstated the fluence rate in the near-lamp region and underestimated it near the walls. Nevertheless, Ho^22^ concluded that the experimental measurements were perfectly matched with the radiation distribution, derived from the DO model by taking into account the quartz walls' reflection and refraction, as well as the impact of reflection from the UV reactor's inner surfaces. UV disinfection performance with different lamp configurations was explored by Xu et al.^23^ using pathogen log reduction under steady UV dose. The findings showed that even though the UV fluence rate distribution was more uniform, increasing the number of lamps had no influence on reactor performance. Li et al.^24^ carried out an investigation on the influence of water UV transmittance (UVT), lamp power, and flow rate on the UV dose distribution and the average dose in a U-shaped reactor. The results indicated that as the lamp power increased and flow rate decreased, the UV dose distribution profiles switched to a higher range of UV dosage, and when UVT increased, the fluence rate grew exponentially. In addition, lower UVT values need a lower flow rate to allow for a more extended particle residence time, whereas higher UVT values necessitate a higher flow rate^5^. Stainless steel is the most utilized material in UV reactors, and it reflects just a small part of the received light^25^. In practice, numerous models have failed to account for the reflection of reactor walls, resulting in less accurate predictions. Furthermore, the near-wall area of most UV reactors has the lowest radiation intensity, significantly limiting total reactor performance^26^. Therefore, one strategy to increase the performance of UV reactors is to use materials that can minimize the loss of UV energy. The impact of the aluminum reflector in an annular photoreactor was investigated by Yang et al.^27^ using a mathematical model. They reported that the light intensity at the reactor's core might be increased by 40% using an aluminum reflector. To enhance the performance of solar disinfection, Navntoft et al.^28^ employed an aluminum reflector. Imoberdorf et al.^29^ used the Monte Carlo radiation model to show how inner wall reflection affects the radiation intensity distribution. Chen et al.^30^ developed a closed-conduit reactor and studied the impacts of wall reflection on water disinfection. The findings revealed that when UVT is low, it is acceptable to ignore the wall reflection effect; however, when UVT is high, the reflection must be considered. Li et al.^31^ considered aluminum foil, stainless steel, and black cloth as inner wall materials to analyze the influence of reflection on fluence distribution. The findings demonstrated that an inner wall with a rough surface that improves diffuse reflection might result in better dose distribution. Also, CFD models were used by Li et al.^32^ to assess the influence of inner wall reflection in annular single-lamp UV reactors, with a particular emphasis on diffuse reflection. The results revealed that the calibrated DO radiation model can be used to correctly estimate the radiation distributions in UV reactors with a reflecting inner wall. In addition, fluence rates were much greater in UV reactors with high reflecting inner walls than the conventional ones, particularly at high water UVTs. Also, the homogeneity of the radiation distribution was improved by the inner wall diffuse reflection. Heidarinejad et al.^25^ carried out a CFD simulation to evaluate the effect of reflecting materials on the performance of a cross-flow UV reactor. They reported that with increasing UVT and lamp power and decreasing flow rate, the impact of reflection on reactor performance increases if the inner wall is constructed of aluminum. Moreover, when the lamp distance from the inner wall is reduced, the impact of reflection becomes more noticeable. To evaluate the effect of surface reflection on microbial inactivation, a model for studying UV-C LED air treatment ducts was designed by Thatcher and Adams^33^. They reported that the microbial inactivation was greatly boosted when highly reflecting surfaces were used, while the influence of LED locations on inactivation levels was limited. A recent study was conducted by Kooshan et al.^34^ to investigate the effectiveness of cylindrical UV-LED photoreactors for water disinfection that are composed of UV reflecting materials analyzing three common configurations. The results showed that the U- and S-shaped configurations perform better than the L-shaped photoreactor using reflecting surfaces.

The comprehensive literature review on the water UV disinfection reactors reveals that they are made of a cylindrical shell with one or more interior quartz cylindrical sleeves containing UV lamps. Water flows between the quartz sleeve and the reactor wall. Such a conventional design includes at least one of the following disadvantages: (1) restricted performance due to minerals, organics, and other particles and compounds, especially at low water UVTs, (2) reduced disinfection dose caused by quartz fouling, (3) overheated UV lamps in low flow rates, (4) broken quartz during lamp replacement. In this study, a novel configuration with a reflective inner wall is presented, with UV lamps positioned between the water route channel and the UV reflector’s inner walls. Few studies have been conducted on LED-based reactors, in which the LED is located outside the primary chamber and is not in touch with fluid^35,36^. The current proposed configuration has significant advantages over typical designs. These include low operating cost, no operator exposure, and quick installation and lamp replacement. We simulate the operation of such reactors with CFD to investigate the effect of several parameters on their performance. The main objectives of this study are summarized as:

- Proposing a new reflection-based geometry for water UV disinfection reactors
- CFD simulation of the proposed system
- Comparing the log reduction value (LRV) in various conditions through the parametric study: number of UV lamps, diffuse fraction, the distance of UV lamps to the water channel, and wall reflectivity of the reactor
- Comparing the LRV of the presented configuration with conventional designs

## Methods

### UV reactor

**Figure 1:**
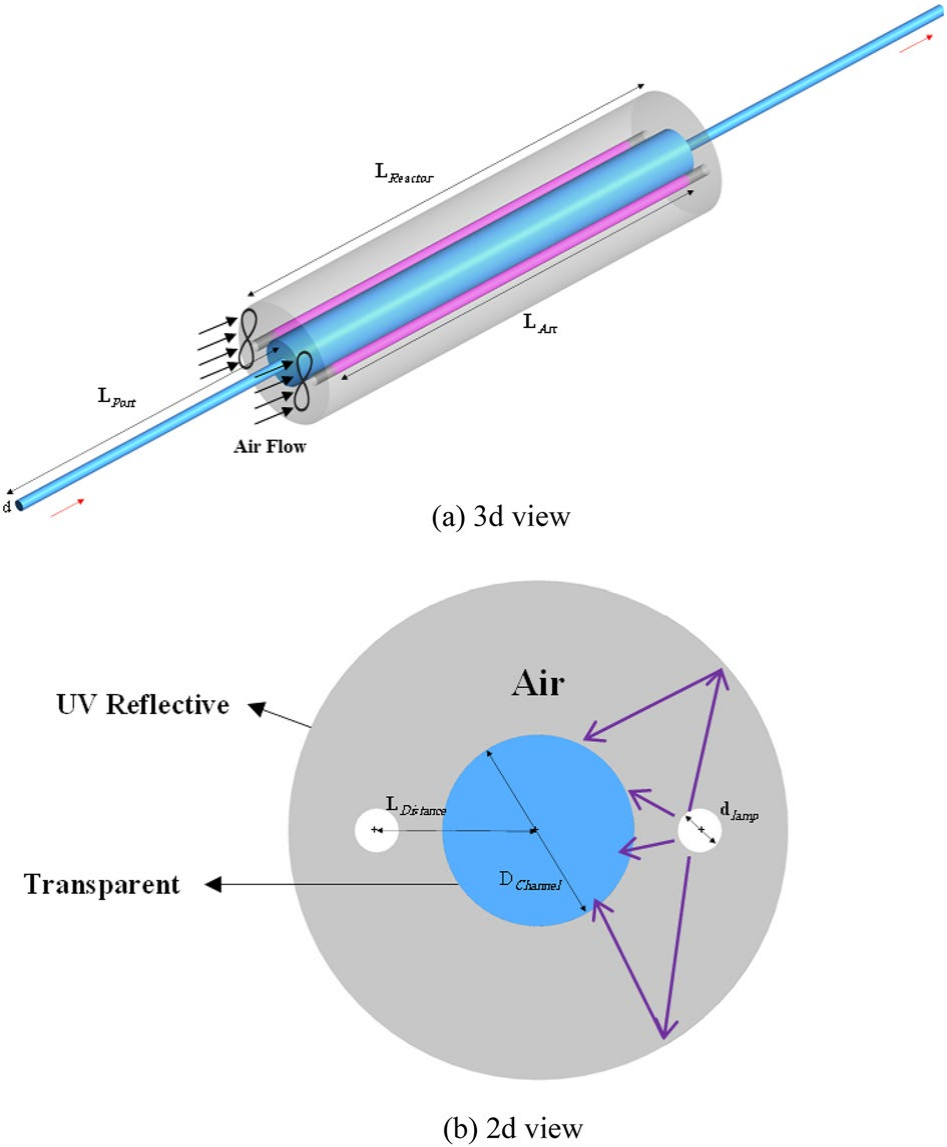
Schematic representation of the novel UV photoreactor for water disinfection.

In this research, a novel tubular UV reactor is being investigated. The diameter of the central tube through which water flows is determined such that the average velocity of water flow within the reactor equals the average velocity of the flow in the annular reactor studied by Sozzi and Taghipour^19^ for the same flow rates. The geometry of the reactor is shown in Fig. 1. This novel design consists of two concentric cylinders; the inner is made of a transparent material with very low UV absorption such as quartz and the outer is made of a powerful UV reflective material such as aluminum or polytetrafluoroethylene (PTFE). A number of UV lamps (two lamps in Fig. 1) are located in the space between the two tubes, which is filled with air. A forced-convection cooling mechanism can be employed to cool down the lamps with the flow of air. In addition, there are inlet and outlet ports in the geometry to make water flow through the reactor. In this design, the UV emitted from the lamps can enter the quartz water channel either directly or after reflection from the outer UV reflective cylinder. Table 1 lists the dimensions of the reactor described. The reactor's length and the lengths and diameters of the UV lamps are identical to the specifications introduced by Sozzi and Taghipour^19^. These choices are made so that we can do a fair comparison between the new design and the conventional ones. The inlet and outlet ports are considered long enough to ensure fully-developed flow in the pipes.

**Table 1:** Specifications of the proposed photoreactor.

Variable	Explanation	Dimension (cm)
${L}_{\text{Re}actor}$	Length	88.9
${L}_{Arc}$	UV arc length	80
${L}_{Port}$	Length of inlet/outlet ports	60
${L}_{Dis\text{tan}ce}$	Length of water channel to UV lamp distance	7.5
${D}_{Channel}$	Water channel diameter	8.672
${d}_{lamp}$	UV lamp diameter	2
$d$	Inlet/outlet pipe diameter	2

### Governing equations

As previously stated, the flow hydrodynamics is crucial within UV water disinfection photoreactors since the flow field directly impacts the residence time of particles and thus it can change the amount of received UV dose among microorganisms. Employing the Reynolds Averaged Navier–Stokes (RANS) approach for the modeling of incompressible and isothermal turbulent flows within a photoreactor, the conservation of mass and momentum equations can be expressed as:

1 $$\frac{\partial {U}_{j}}{\partial {x}_{j}}=0$$

2 $$\frac{{\partial (U_{i} U_{j} )}}{{\partial x_{j} }} = \frac{{ - 1}}{\rho }\frac{{\partial p}}{{\partial x_{i} }} + \frac{\partial }{{\partial x_{j} }}\left( {\nu \frac{{\partial U_{i} }}{{\partial x_{j} }} - \overline{{u_{i} u_{j} }} } \right),$$

where ${U}_{j}$ denotes the components of time averaged velocity vector, $p$ is the pressure, $\nu$ is the kinematic viscosity, and $-\rho \overline{{u}_{i}{u}_{j}}$ is the components of the Reynolds stress tensor, which must be related to the components of the averaged velocity vector by employing the Boussinesq assumption and a valid RANS turbulence model. In this work, the flow field in UV reactors is simulated using the realizable *k*–*ε* turbulence model^37^, which has shown enough accuracy for the flow fields of the same type^18^.

The DO model is employed to obtain radiation intensity within the computational domain. This model has proved to be successful for different geometries of UV reactors as mentioned in several studies^24,25,38,39^. For non-gray radiation modeling, the radiation transport equation (RTE) is solved for an arbitrary number of solid angles at control volumes and a single wavelength $\lambda$ in this model^40^. The transport equation can be described as follows:

3 $$\nabla \cdot \left( {I_{\lambda } (\vec{r},\vec{s})\vec{s}} \right) + \left( {a_{\lambda } + \sigma _{s} } \right)I_{\lambda } \left( {\vec{r},\vec{s}} \right) = a_{\lambda } n^{2} I_{{b\lambda }} + \frac{{\sigma _{s} }}{{4\pi }}\int\limits_{0}^{{4\pi }} {I_{\lambda } \left( {\vec{r},\vec{s}} \right)\Phi \left( {\vec{s} \cdot \overrightarrow {{s^{\prime}}} } \right)d\Omega ^{\prime}} ,$$

where $\overrightarrow{r}$ is the position vector, $\overrightarrow{s}$ is the direction vector, $\overrightarrow {{s^{\prime } }}$ is the scattering direction vector, $n$ is the refractive index, ${\sigma }_{s}$ is the scattering coefficient, ${I}_{b\lambda }$ is the black body intensity, ${I}_{\lambda }$ is the radiation intensity, $\Phi$ is the phase function, Ω′ is the solid angle, and ${a}_{\lambda }$ is the absorption coefficient. It should be noted that the absorption coefficient can be linked to UVT of water through Beer–Lambert’s law^41^:

4 $$UVT={e}^{-ax}$$

where $x$ [cm] is the distance between two points in the medium.

The inactivation of microorganisms in the UV disinfection process is directly linked to the amount of UV dose absorbed:

5 $$D={\int }_{0}^{t}Idt,$$

In Eq. (5), $D$ is the amount of absorbed dose, $I$ is the radiation intensity, and $t$ is the residence time of pathogens within photoreactor.

The amount of inactivation is typically measured by log reduction value (LRV), which is computed as the logarithm of the ratio of initial active microorganisms to the instantaneous number of active microorganisms. The rate at which microorganisms are inactivated is a function of the overall UV dose absorbed. Therefore, LRV can be written as:

6 $$LRV=\text{log}\left(\frac{{N}_{0}}{N}\right)=f\left(D\right),$$

In this work, we have chosen MS2 (*E. coli* bacteriophage ATCC 15597-B1) virus as the water contaminator pathogen.

The survival ratio of microorganisms, indicated by ${R}^{*}= N/{N}_{0}$, can be stated as follows in the Eulerian framework^17^:

7 $$\frac{\partial }{\partial {x}_{j}}\left({U}_{j}{R}^{*}\right)=\frac{\partial }{\partial {x}_{j}}\left({D}_{eff}\frac{\partial {R}^{*}}{\partial {x}_{j}}\right)+S,$$

where ${D}_{eff}$ is the effective diffusion coefficient, $S$ is the volumetric rate of microorganisms destruction in the medium, and ${U}_{j}$ is the velocity vector computed from the modeling of flow field. The effective diffusion coefficient is stated as ${D}_{eff}={\nu }_{t}/ S{c}_{t}$ considering that the molecular diffusion of microorganisms in water is negligible^17^. With reference to the literature^42^, Schmitt number of $S{c}_{t}=0.7$ is proposed for flow in cylindrical conduits. The sink term in Eq. (7) may be found by utilizing the chain rule differentiation to both sides of Eq. (6) with respect to time:

8 $$S=\frac{d{R}^{*}}{dt}=\frac{d}{dt}\left(\frac{N}{{N}_{0}}\right)=-\text{ln}\left(10\right){R}^{*}I\frac{df(D)}{dD}$$

where the inactivation curve of MS2 is given by the following equation:

9 $$f\left(D\right)=9\times 1{0}^{-10}{D}^{3}-3\times 1{0}^{-6}{D}^{2}+0.0062D$$

This equation is similar to the MS2 fluence-response curve used by Elyasi and Taghipour^17^. Note that the derivative of the above relation with respect to dose is dependent on the amount of dose at each point, for which an inverse function was given by Elyasi and Taghipour^17^ as:

10 $$D=22.264{\text{log}}^{2}\left(\frac{{N}_{0}}{N}\right)+149.6\text{log}\left(\frac{{N}_{0}}{N}\right)$$

by substituting the derivative of Eq. (9) in the sink term, Eq. (8) can be re-written as follows:

11 $$S=\frac{d{R}^{*}}{dt}=\frac{d}{dt}\left(\frac{N}{{N}_{0}}\right)=-\text{ln}\left(10\right){R}^{*}I(27\times 1{0}^{-10}{D}^{2}-6\times 1{0}^{-6}D+0.0062)$$

where $D$ is calculated using Eq. (10). Both Eqs. (9) and (10) are defined in ANSYS-Fluent using a user-defined function.

### Numerical methods and boundary conditions

Throughout this work, the modeling procedures have been carried out using ANSYS-Fluent commercial CFD package. ANSYS-Fluent is an unstructured finite volume flow solver that uses the cell-centered approach to discretize and solve equations on collocated grids. Finite volume methods have proven to be applicable to a wide range of flow problems and can be used in adaptive frameworks^43,44^. In our work, the Reynolds number in the water channel ranges between 40,000 and 100,000 for various flow rates, which is well beyond the transition limit of internal flows. Considering that the flows are fully turbulent, we employ the realizable *k*–*ε* turbulence model to provide the closure for the system of equations. To make the cost of simulations reasonable, the standard wall functions is utilized for the treatment of near wall regions. The equations of the incompressible flow field are solved using the SIMPLE pressure–velocity coupling technique. The least-squares approach is used for cell gradient calculation; the convective terms of flow equations are discretized using a second-order upwind scheme, which has known accuracy advantages^45^. The walls are treated with the no slip boundary condition. The averaged velocity obtained from the volumetric flow rate of interest is imposed at the inlet; the atmospheric pressure is set for the outlet of the reactor. The turbulence intensity of 5% is prescribed at the reactor's inlet.

To simulate the radiation field, the UV sources are regarded as low-pressure mercury lamps that generates UV radiation with the wavelengths of 254 nm and the total germicidal output power of 35 W. This is the output power of the single UV lamp of the photoreactor studied by Sozzi and Taghipour^19^. It should be noted that in multi-lamp arrangements, the total power is equidistributed between the lamps. For example, the effective power of each UV source in a two-lamp arrangement is 17.5 W. The simulations are performed with water and air as working fluids. It is worth mentioning that the flow of air is not modeled here as it has no effect on the distribution of radiation intensity within the water channel and thus reactor’s performance. The absorption coefficients of water and air is considered as 35.66 and 0 $[1/m]$, and their refractive indices is set as 1.36 and 1.003, respectively. The obtained absorption coefficient of water corresponds to a $UVT$ of 70%. Water typically has $UVT$ of more than 70%, but in some cases, when water contains UV-absorbing elements, $UVT$ can drop as low as 70%. So, water $UVT$ of 70% can be considered as the worst-case scenario^13,24^. It is also assumed that the water channel wall within the reactor is fully transparent, causing no refraction or scattering of radiation beams. For the UV reflective surface of the reflector, the following relations can be used for the evaluation of emission, reflection, and absorption at the walls^46^:

12 $$\text{wall emission}={n}^{2}{\varepsilon }_{w}\sigma {T}_{w}^{4}$$

13 $$R=\left(1-{\varepsilon }_{w}\right)$$

14 $$\text{wall diffuse reflection}={f}_{d}R{q}_{in}$$

15 $$\text{wall specular reflection}=\left(1-{f}_{d}\right)R{q}_{in}$$

16 $$\text{wall absorption}={\varepsilon }_{w}{q}_{in},$$

where $R$ is the wall reflectivity, ${f}_{d}$ is the diffuse fraction, $n$ is the refractive index, ${\varepsilon }_{w}$ is the internal emissivity, $\sigma$ is the Stephen–Boltzmann constant, ${q}_{in}$ is the total energy, and ${T}_{w}$ is the wall temperature. As a result, by adjusting the ${\varepsilon }_{w}$ and ${f}_{d}$ values, several inner-wall reflection scenarios can be attained. Reflective behavior can be classified into two types: diffuse reflection and specular reflection. When incoming radiation is re-scattered by the surface molecules, diffuse reflection occurs. This sort of reflection reflects light in all directions, independent of the angle at which it is impacted. Lambertian surface is the name given to a purely diffused surface. Specular reflection happens at the surface's contact, reflecting light in certain directions that are dependent on the angle of incident light. Mirrors may be thought of as near-perfect representations of pure specular surfaces^47^. In our work, we have used ${f}_{d}$ values of 1, 0.5, and 0, which model purely diffused, half-specular, and purely specular walls. It is also assumed that the reflectivity of the UV reflective surface of the reflector was set to 95%^48^.

The energy and radiation transport equations are discretized using second-order upwind and first-order upwind schemes, respectively.

For the transport equation of pathogens’ concentration, the survival ratio of one is prescribed at the inlet, suggesting that all the microorganisms are active prior to disinfection. The mass flow of zero is implemented at the walls and outlet of the reactor. The convection–diffusion equation of pathogens concentration is discretized using the third-order MUSCL method. All of the governing equations’ normalized residuals are set to ${10}^{-5}$ as the convergence criterion except for the transport equation associated with pathogen concentration, Eq. (7), where the convergence is obtained at the residual of ${10}^{-6}$.

### Domain decomposition

**Figure 2:**
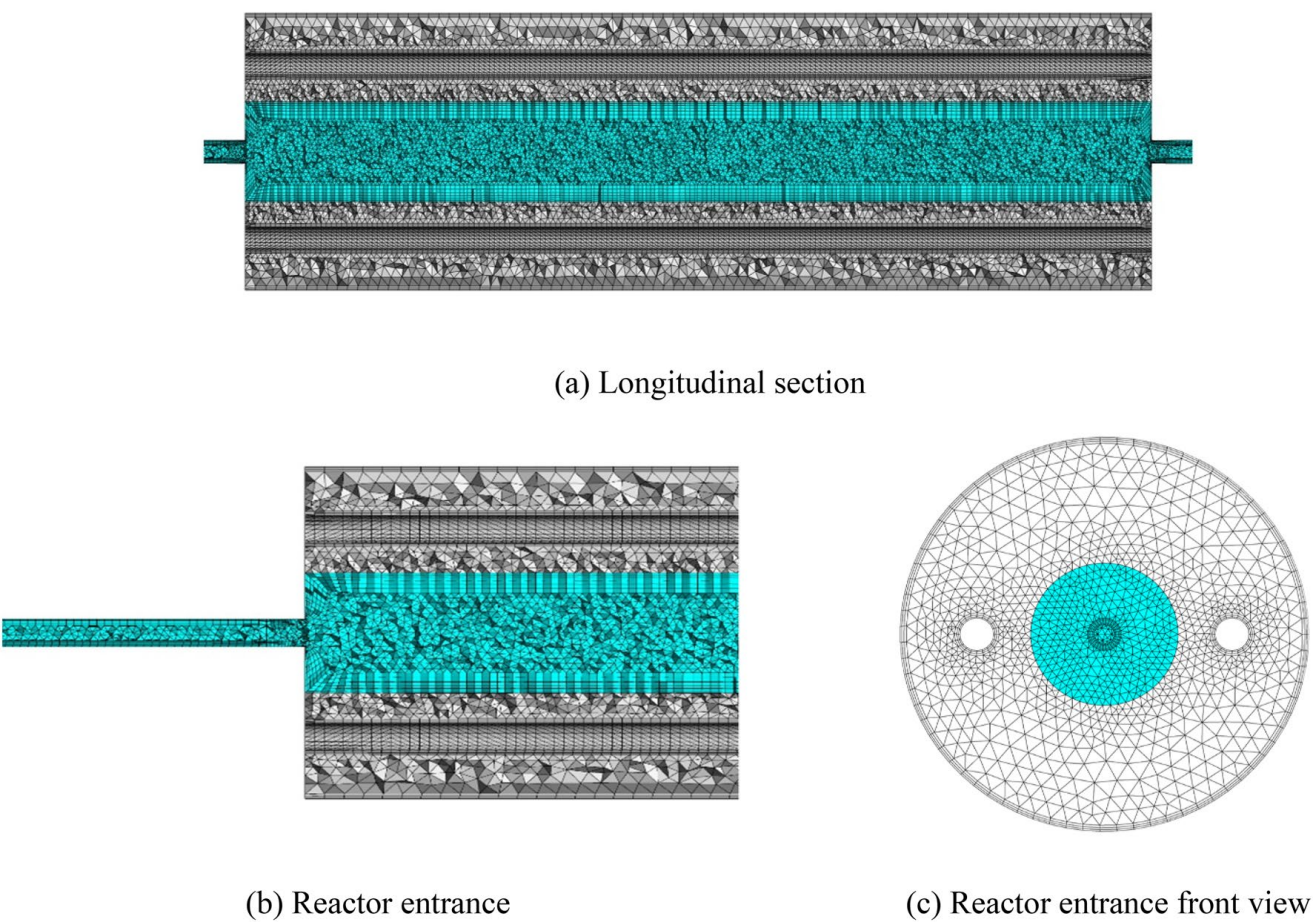
Different views of the unstructured mesh used for CFD simulations.

In the current study, different unstructured meshes have been produced using the ANSYS-meshing software. The majority of the computational domain is covered by tetrahedral cells, while flow characteristics near the walls are captured by boundary layer mesh comprised of stretched prisms. The boundary layer mesh is obtained by the stretching factor of 1.2 on six layers. Due to the use of the wall functions in these locations, the first layer of the boundary layer mesh is placed such that ${y}^{+}=50$. Figure 2 shows the final created grid for the flow rate of 25 US gallon per minute (GPM).

**Figure 3:**
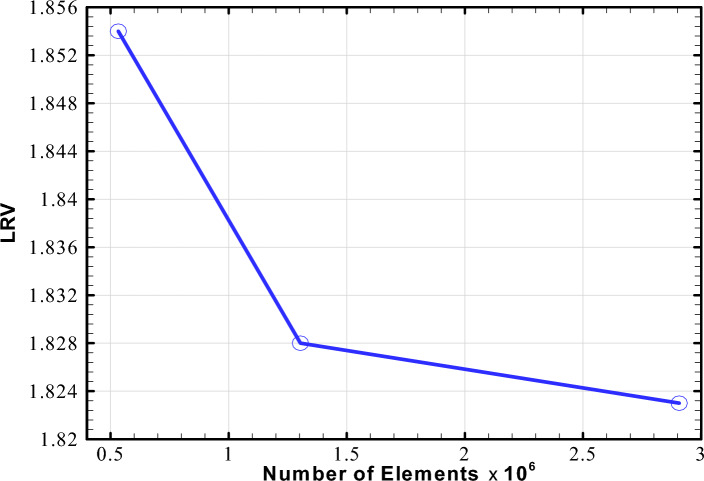
Mesh independence study for the CFD simulation of novel UV photoreactor at the flow rate of 25 GPM.

The area-weighted average of LRV at the reactor outlet was adopted as the output parameter to evaluate the influence of mesh spacing on the numerical findings. Figure 3 illustrates the variation of LRV at the outlet with respect to the mesh size. The grid convergence index (GCI) was computed based on the value of output on three different meshes with 533,000, 1,304,000, and 2,907,000 cells for the flow rate of 25 GPM. The procedure described by Celik et al.^49^ was utilized and the GCI value of 0.1064% was obtained. This suggests that the mesh with 1,304,000 cells is fine enough to produce grid-independent results. The same procedure was employed for the flow rates of 10, 15, and 20 GPM, and enough resolution was obtained for each case. Hence, the unstructured meshes with approximately 1,180,000, 1,246,000, 1,276,000, and 1,304,000 cells have been obtained for the four flow rates of 10, 15, 20, and 25 GPM, respectively. In addition, the independence of radiation field solution from the solid angles was investigated in this research using three different angular discretizations. The angular discretization was set to $4\times 4,$ $6\times 6,$ and $8\times 8,$ respectively, and the fluence rate distribution was compared at the mid-plane of the reactor for two radial lines in the x- and y-directions, respectively (see Supplementary Fig. S1 online). The results show that the largest difference between the angular discretization of $4\times 4$ and $8\times 8$ is 3.2%, while the maximum difference between the angular discretization of $6\times 6$ and $8\times 8$ is 0.7% in the x-direction. Similarly, with respect to the angular discretization of $8\times 8$, the angular discretization of $4\times 4$ and $6\times 6$ can differ by up to 3% and 0.9%, respectively, in the y-direction. Hence, the angular discretization of $6\times 6$ is precise enough to yield findings that are independent of solid angles.

## Model validation

**Figure 4:**
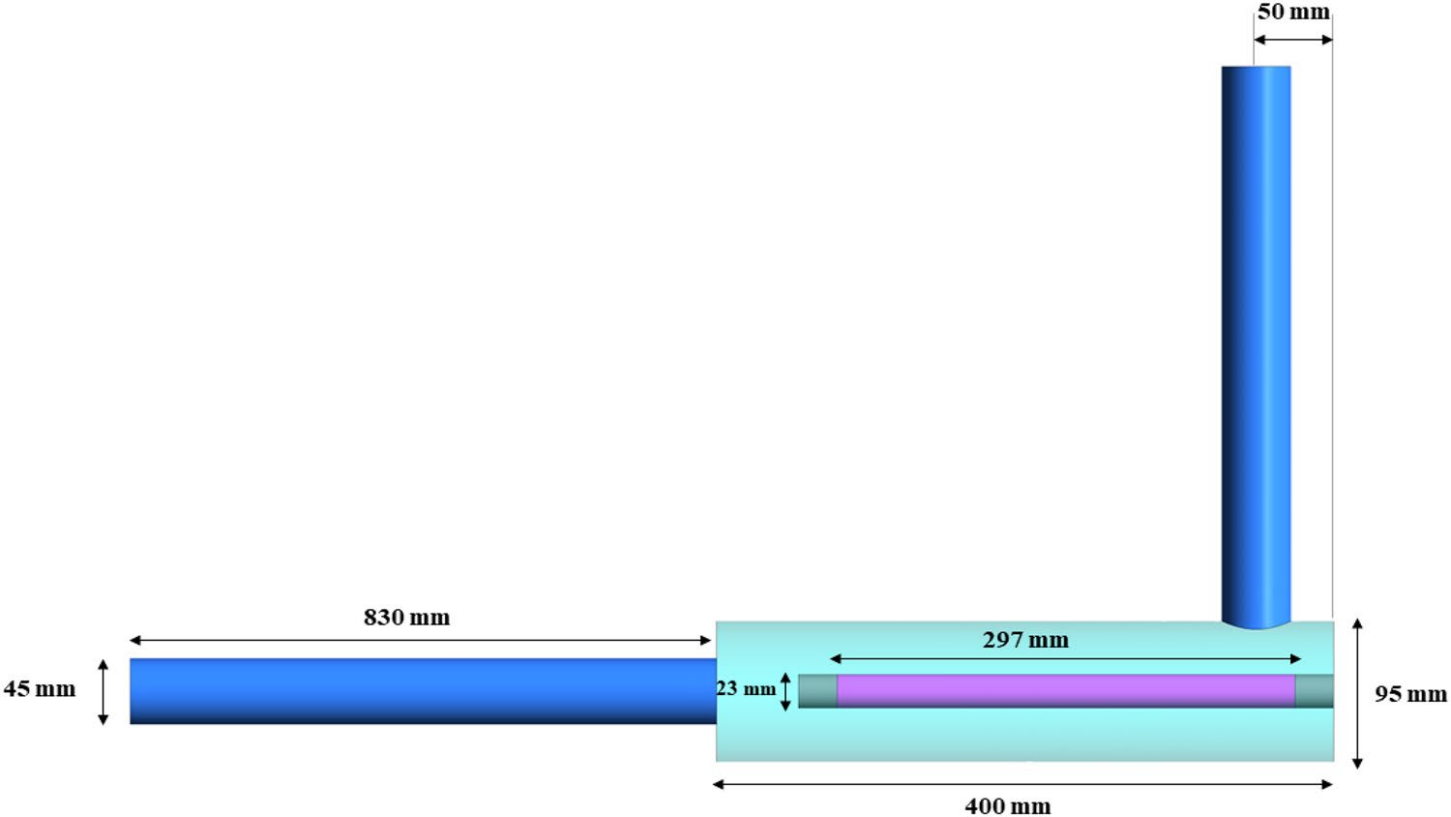
Schematic representation of the validation case.

To ensure about the correctness of our CFD model, we compare the outputs of our model with the experimental data available in the literature for a UV photoreactor having different geometry but working based on reflection from the walls. In this way, we can validate the distribution of UV fluence rate obtained by our model and also the amount of microbial inactivation. For this purpose, we chose the photoreactor studied by Li et al.^32^. This validation case is in the shape of traditional L-shaped photoreactors, where water enters from the center of one end and exits from a port located on the outer surface of the main body near the other end. The diameter of the inlet/out ports and main body are 45 and 95 mm, respectively; the length of the inlet/out ports and main body are 830 and 400 mm, respectively, and the distance between the central axis of the outlet port and the end of the reactor is 50 mm. Figure 4 shows the geometry of validation case along with relevant dimensions. The output power, quartz sleeve diameter, UVC efficiency, and the wavelength of radiation for the low-pressure mercury lamp are 16 W, 23 mm, 26%, and 254 nm, respectively. The lamp arc length is 297 mm while the length of the quartz sleeve is 347 mm. Two types of inner-walls with reflectivities of 0.26, and 0.8 with three different diffuse fractions of 0.1, 0.5, and 0.9 were studied in the original work.

**Figure 5:**
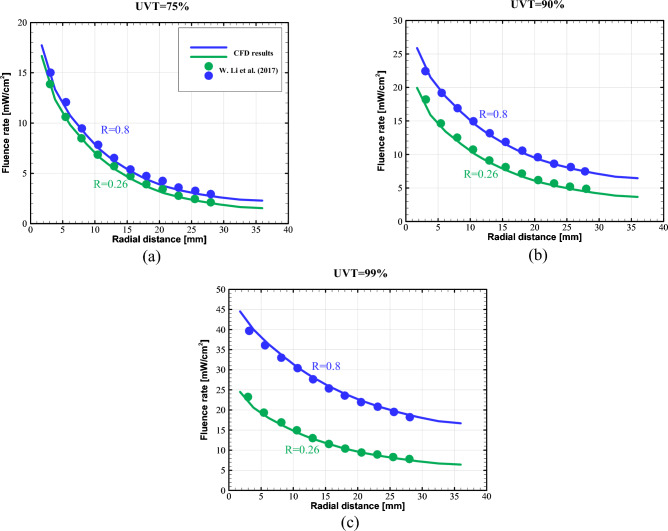
Comparison of radial fluence rate distribution with experimental data in two wall reflectivities for (**a**) UVT = 75%, (**b**) UVT = 90%, and (**c**) UVT = 99%.

For the chosen validation case, Fig. 5 shows the radial fluence rate distributions in the middle planes of UV reactors at different water UVTs with two various wall reflectivities. It should be mentioned that the fluence rate is outlined as the sum of all radiation intensities throughout the entire solid angle. A micro-fluorescent silica detector (MFSD) was developed by Li et al.^50^ to acquire the fluence rate distribution. They employed a Ge-doped silica cylinder specifically as a detector in their research. A chemical vapor deposition technique was used to create the Ge-doped silica, which contained $\left(10\pm 0.5\right)\%$ of GeO_2_. Photons can be captured from all directions by this cylindrical-shaped detector, whose vertical axis was aligned with the lamp. A photodiode was used to detect the fluorescence, which was then amplified and shown on a multimeter after being received by an optical fiber.

As observed, the fluence rate distributions predicted by our model, is in excellent agreement with the experimental data regardless of wall reflectivities.

Considering that the overall performance of a UV photoreactor is dependent on both the fluid flow, and radiation intensity distribution, we need to compare the amount of microbial inactivation with the validation test case. Li et al. considered a first-order kinetic model for the microbial inactivation process, which is given as follows:

17 $$LRV=\text{log}\left(\frac{{N}_{0}}{N}\right)=f\left(D\right)=k(D-{F}_{0})$$

where $k$ is the kinetic rate constant obtained from experimental data, and ${F}_{0}$ is the intercept of the fluence-response curve for *Bacillus subtilis.* The values of $k$ and ${F}_{0}$ were set to 0.087 (cm^2^/mJ) and 3.0 (mJ/cm^2^), respectively. For this first-order kinetic model, the sink term in Eq. (7) can be expressed as follows:

18 $$S=\frac{d{R}^{*}}{dt}=\frac{d}{dt}\left(\frac{N}{{N}_{0}}\right)=-ln\left(10\right){R}^{*}Ik$$

**Figure 6:**
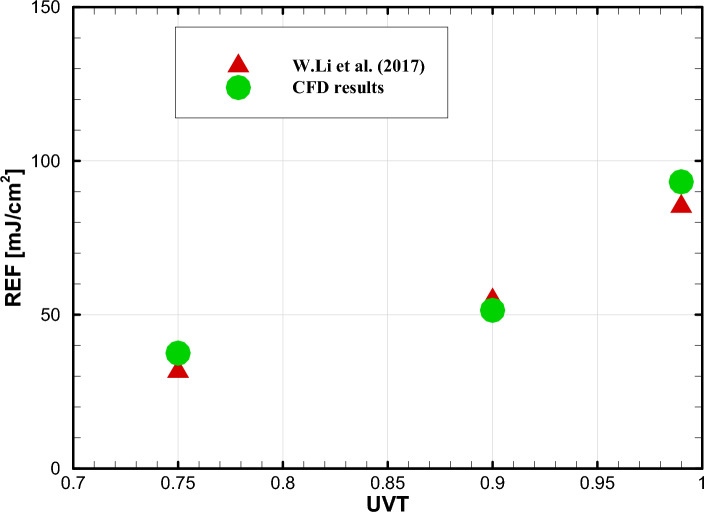
Validation of REF at three different water UVTs.

Figure 6 indicates the reduction equivalent fluence (REF) of the reactor at different water UVTs with inner-wall reflectivity of 0.26. The diffuse fraction is set at 0.1. REF can be defined as radiation equivalent dose (*D* at Eq. (17)) for a specific LRV. The REF can be determined from Eq. (17) once LRV has been obtained by simulating the inactivation process of the microorganisms.

As seen, our simulation results are perfectly in agreement with Li et al. investigations. It is worth mentioning that our simulation results for REF were achieved using the Eulerian method, whereas Li et al. used the Lagrangian method. The small discrepancies can be attributed to this difference.

It should be noted that the flow field in our proposed UV reactor is much simpler than the validation test case, as it includes the turbulent flow in a pipe with sudden expansion. The flow field in our proposed UV reactor is greatly in agreement with 1/7th power law turbulent velocity profile. Considering that the accuracy of our radiation modeling and microbial inactivation was validated, the overall simulation is likewise valid. So, we can move forward and use the CFD model for the performance evaluation of the photoreactor of interest in the following section.

## Results and discussion

Having validated our CFD model, we turn our attention back to the novel UV photoreactor proposed in “Methods” to study the effect of different parameters on its microbial performance. Several variables such as the number of lamps, wall reflectivity and its type, and also reactor dimensions are investigated as follows.

### Effect of number of UV lamps

In this section, three different modes of lamp arrangement are investigated to comprehensively examine the influence of number of UV lamps on microorganism inactivation. For this purpose, the proposed photoreactor of “UV reactor” with two, four, and six UV lamps are considered. The lamps are uniformly placed around the water pipe; their centers are 7.5 cm apart from the center of water pipe. It should be noted that these cases provide distinct radiation distributions but identical flow fields since there is no interaction between the path of water and UV lamps.

**Figure 7:**
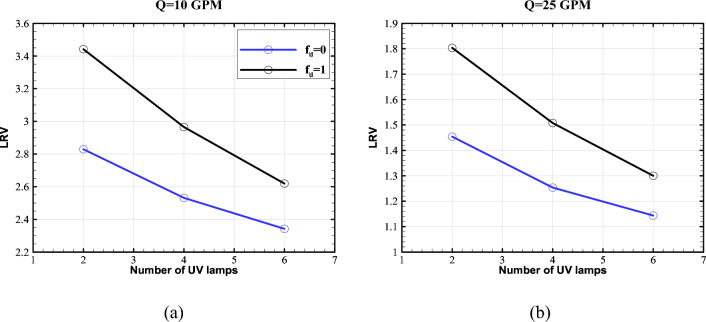
Proposed reactor's LRV versus the number of UV lamps at the flow rates of (**a**) 10 GPM and (**b**) 25 GPM.

Figure 7 depicts the performance of the proposed UV reactor for different number of lamps at the flow rates of 10, and 25 GPM. To make a meaningful comparison, the overall output power of lamps is kept constant and equal to 35 W over the arc length. This is equal to the output power of the single lamp photoreactor investigated by Sozzi and Taghipour^19^. Moreover, the comparison was performed for the reflectivity of 95% at the two reflection modes of full diffuse (${f}_{d}=1$) and full specular (${f}_{d}=0$). The area-weighted average of LRV at the outlet was used as the performance indicator of each case.

**Figure 8:**
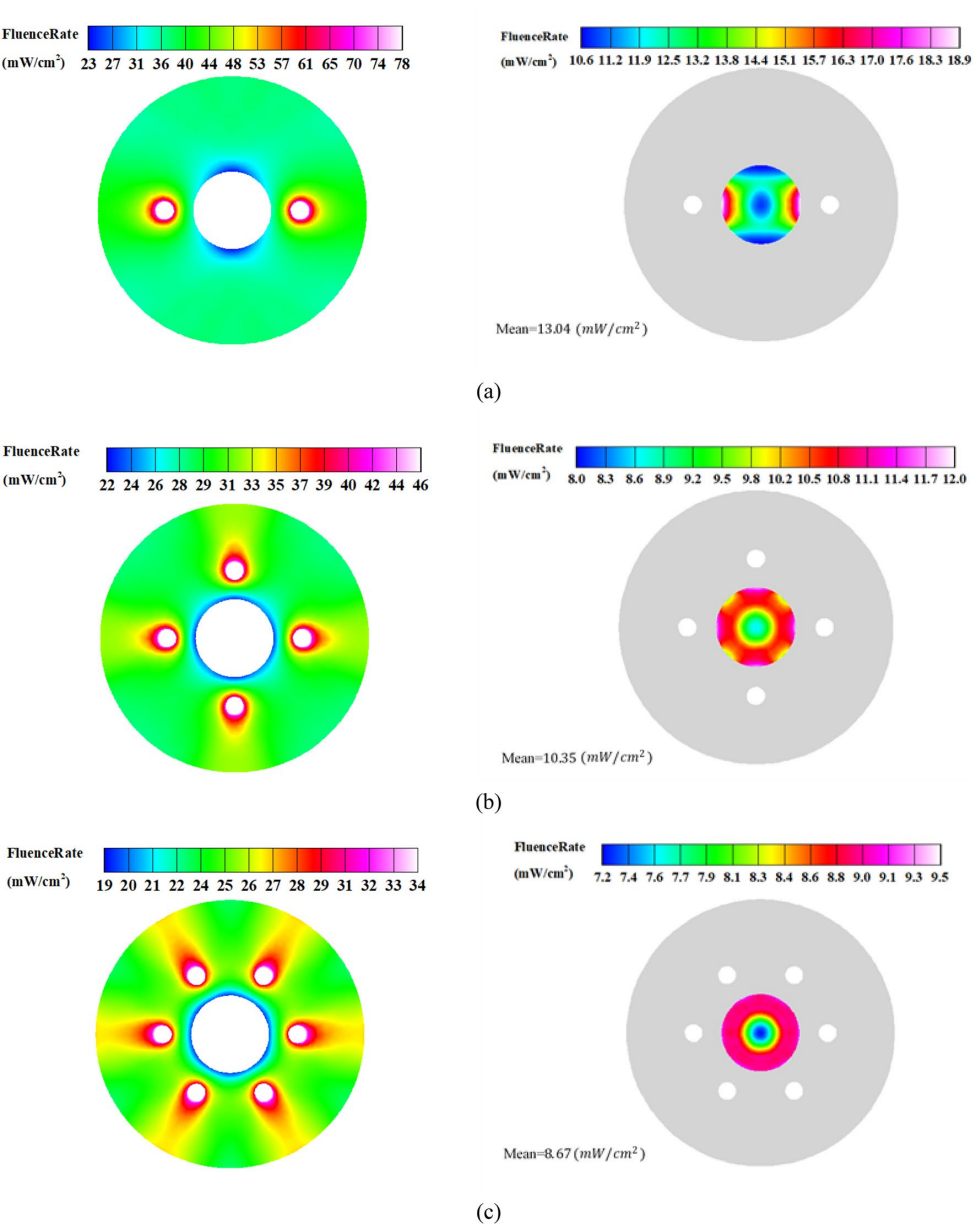
Distribution of Fluence rate at the mid- plane of reactor with fully-diffuse UV reflector for (**a**) 2-lamp, (**b**) 4-lamp, and (**c**) 6-lamp arrangements.

**Figure 9:**
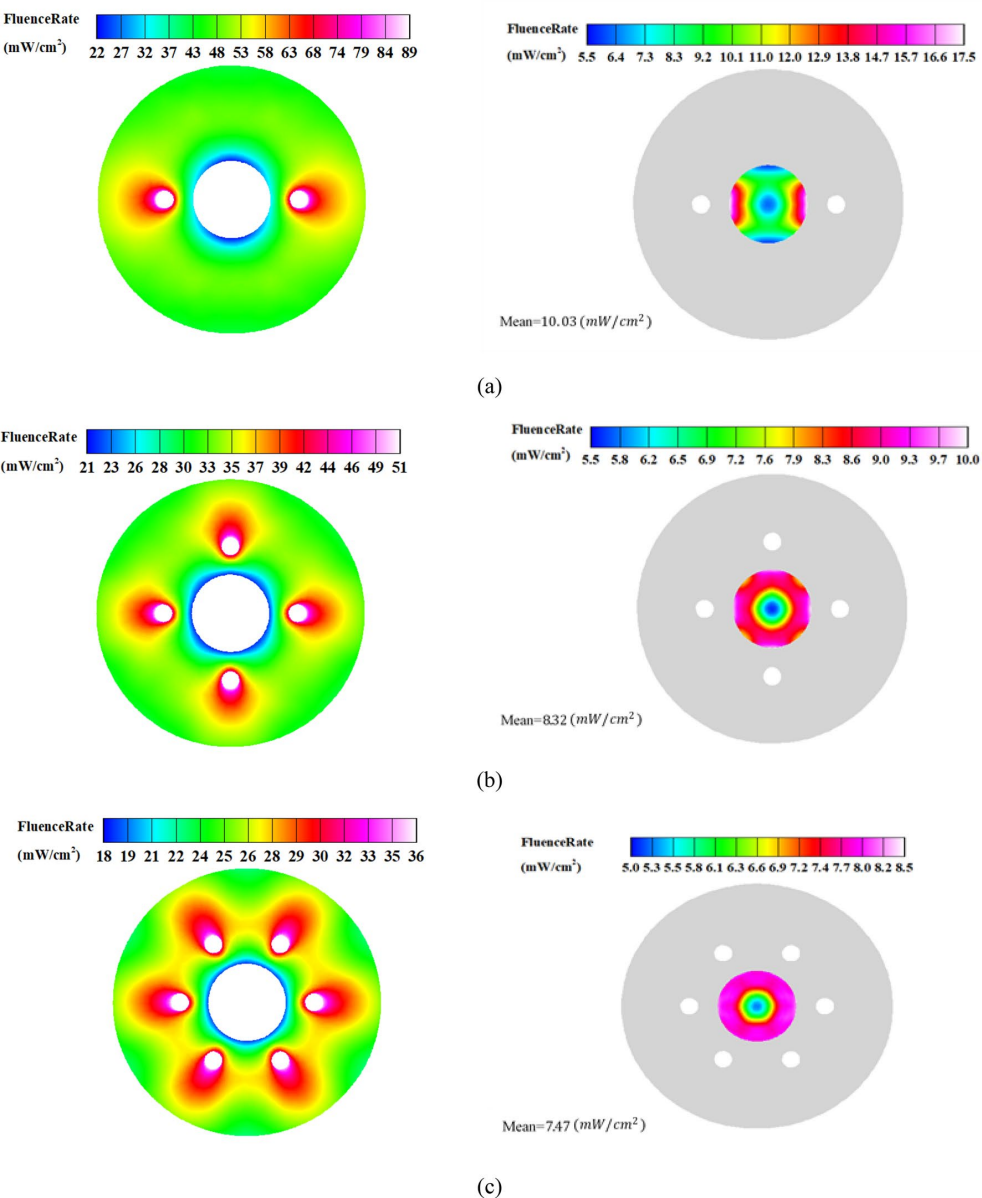
Distribution of Fluence rate at the mid- plane of reactor with fully-specular UV reflector for (**a**) 2-lamp, (**b**) 4-lamp, and (**c**) 6-lamp arrangements.

As seen, the reactor with two lamps outperforms the other configurations, and there is a clear trend of LRV decrease as the number of UV lamps increases for both the diffuse and specular reflection modes. This can be explained by two reasons. Firstly, the UV intensity in the regions close to the lamps within the water pipe decreases as the power of lamps decreases for larger numbers (the total power is constant). Secondly, the presence of more lamps causes a shadowing effect, which reduces the number of reflected beams from the outer cylinder incident on the water pipe. This can be understood by the fact that the effective area for the transmit of UV beams decreases as the number of lamps increases. Figures 8 and 9 depict the distribution of UV fluence rate for the diffuse and specular reflection modes, respectively. As observed, the maximum value of the fluence rate in the two-lamp arrangement is considerably higher than the other modes. In fact, the two-lamp arrangement has the potential to enhance the maximum fluence rate by factors of 2.3 and 2.5 for the diffuse and specular reflections, respectively, when compared to the six-lamp arrangement. It should be mentioned that UV lamps absorb ultraviolet energy that either directs or reflects off of their surface. As a result, the fluence rate is decreased in proximity to the water pipe. In addition, some UV photons that reach the surface of the water chamber are reflected and do not enter the chamber because of the difference in refractive indices of the two sides of the surface. Also, even while the distribution of fluence rates for the reactor with four and six lamps is more uniform than that of the reactor with two lamps, the two-lamp mode performs better due to its higher average fluence rate in the water channel. This illustrates that the fluence rate's average value in the water channel has a more important impact than the fluence distribution's homogeneity.

Another conclusion from Fig. 7 is that when the diffuse fraction increases, so does LRV. However, the effect of the diffuse fraction increase is more significant for the two-lamp arrangement. As the number of the lamps increases, the difference in LRV between the fully diffuse and fully specular reflection modes decreases. This can also be explained by the shadowing effect. It is known that incident beam to a reflector is evenly reflected at all different angles in the fully diffuse mode. As can be seen, the reduction of LRV caused by the reduction of diffuse fraction is stronger than the effect of increasing the number of UV lamps.

### Effect of diffuse fraction

In this section, three different diffuse fractions are chosen to study the impact of reflection type on pathogen's inactivation for two flow rates. The diffuse fractions of 0, 0.5, and 1 are considered, which correspond to the fully specular, an equally diffuse-specular, and the fully diffuse reflections, respectively. For this study, different diameters of UV reflector are examined. These diameters are reported in Table 2 for each diffuse fraction. The objective for this is to capture the accurate trend of each reflection type. Therefore, as listed in Table 2, the diameters for the reflection types are carefully selected to demonstrate the pattern of changes. As noticed, a wider range is needed for the half-specular and fully-specular modes.

**Table 2:** Values of reflector diameter considered for different wall reflection modes.

Conditions	Diameter values (cm)
${f}_{d}=1$	[18, 24, 30, 36, 42]
${f}_{d}=0.5$	[18, 30, 42, 54, 66]
$${f}_{d}=0$$	

**Figure 10:**
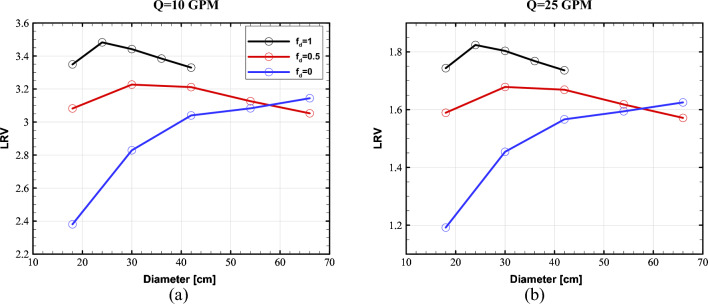
UV reactor performance with different diffuse fractions at various diameters for the flow rates of (**a**) 10 GPM and (**b**) 25 GPM.

Figure 10 demonstrates the LRV of the two-lamp UV reactor for various diffuse fractions with wall reflectivity of 95%. As shown in Fig. 10, when the wall reflection is fully diffuse, LRV initially ascends to a maximum value, then drops as the reflector's diameter increases. Also, the fully diffuse reflection trend may be adequately obtained at small diameters. A possible explanation for this is that the diffusely reflected UV beams are incident to the water channel in all directions. Therefore, smaller diameters and thus shorter travel distance enhances the possibility of UV ray absorption by the contaminated water.

However, a clear rising trend in LRV is seen for the fully specular mode as the reactor diameter increases. This upward tendency can be attributed to the fact that as the diameter of the reactor increases, the blocking effect of the UV lamps reduces, and the water channel is exposed to more reflected rays. Consequently, more UV radiation penetrates into the polluted water, and LRV continues to increase. UV rays are reflected as the reactor diameter increases until they are eventually absorbed by the contaminated water.

As shown in Fig. 10, the half-specular reflection combines the characteristics of both the fully specular and fully diffuse reflection. In particular, it shows a similar trend to the fully diffuse reflection model while a wide range of reactor diameters are necessary to capture the behavior.

The comparison of the three models of reflection reveals that LRV of fully diffuse and half-specular modes differ substantially from that of pure specular types (see Fig. 10). However, at the largest diameter, LRV of the fully specular wall exceeds LRV of the half-specular wall. Nevertheless, the fully diffuse reflection mode provides the greatest performance. In addition to having a higher average fluence rate than the other modes, the completely diffused mode also exhibits more homogeneity in the number of equal lamps (see Supplementary Fig. S2 online). Thus, it comprises two components that improve the effectiveness of the disinfection. This observation is consistent with the findings reported by Li et al.^32^. A uniform fluence rate distribution has been historically regarded as a performance booster for UV reactors^51^. As a result, increasing the diffuse reflectivity of the reactor wall is recommended for the proposed reactor to increase the performance. Note that previous studies have not typically recommended increasing the wall diffuse fraction by means of more roughness due to the additional wall fouling^32^. However, there is no contact between the wall reactor and the contaminated water in the studied reactor of this paper.

Another point noticed in Fig. 10 is the considerable difference in the range of LRV between the two flow rates. This is also expected as decreasing the average velocity (volumetric flow rate) of polluted water going through the water channel increases the residence time of pathogens inside the reactor. Therefore, higher UV dose is received and larger LRVs are attained. It is noteworthy that the variation of LRV for the three modes of reflection is unchanged.

### Impact of lamp-to-channel distance

**Figure 11:**
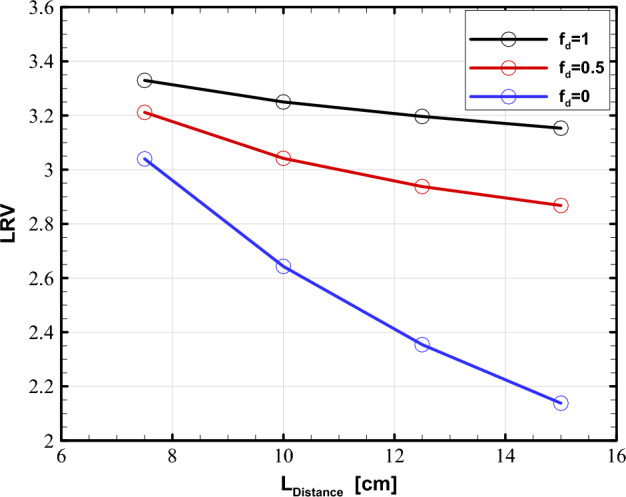
Effect of lamp-to-channel distance on LRV for various diffuse fractions at the flow rates of 10 GPM.

As shown in Figs. 7 and 10, our results follow the same pattern for both flow rates. Therefore, the effect of the distance of the UV lamp from the water channel is studied only for the flow rate of 10 GPM; the reflector’s diameter is considered to be 42 cm. The UV reactor's performance at various lamp-to-channel distances is depicted in Fig. 11 for different modes of wall reflection.

As the UV lamps move farther from the channel, the LRV drops for all three reflection modes. The observed decrease in LRV may be attributed to the fact that as the distance between the UV lamps and the water chamber increases, more beams collide with the reflector’s wall rather than entering the water channel directly, causing part of their energy to be lost. Consequently, the contaminated water receives less intense UV irradiation, and thus LRV drops. Furthermore, when the distance between the UV lamps and the water chamber increases, the LRV variation across the three reflection modes widens, as the fully specular mode has the highest drop. Hence, it can be argued that the influence of lamp-to-channel distance on LRV becomes increasingly substantial as the diffuse fraction diminishes. This is also expected as the reflected beam from a fully specular surface travels in a specific direction that may not enter the water channel. On the other hand, the fully diffuse reflector reorients the beams in all direction, part of which will surely be incident on the water pipe.

### Impact of wall reflectivity

**Figure 12:**
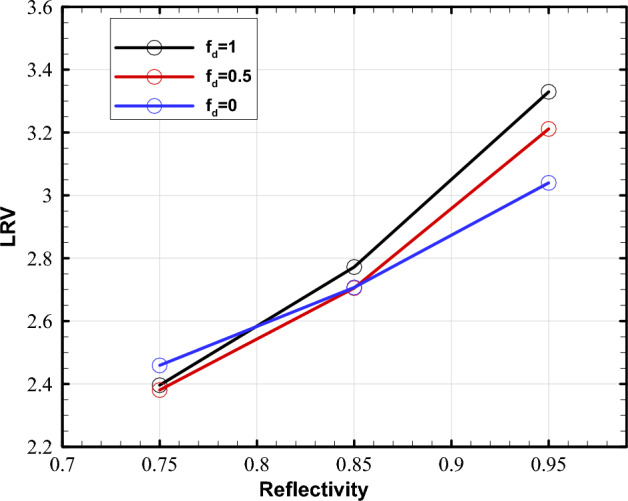
Impact of wall reflectivity on LRV for different diffuse fractions at the flow rates of 10 GPM.

Figure 12 illustrates the LRV versus reflectivity with three different diffuse fractions for the reactor diameter of 42 cm. The flow rate of 10 GPM and the lamp-to-channel distance of 7.5 cm are used since they provided a maximum LRV in the preceding sections. As expected, increasing the reflectivity of the walls causes an upward trend of LRV regardless of reflection type. However, the difference in LRV at the lowest reflectivity (*R* = 0.75) is less significant among the three modes; the difference becomes more considerable as the reflectivity increases. Hence, it can conceivably be concluded that high wall reflectivities significantly contribute to the importance of diffuse fraction on reactor performance. Also, the pure specular type produces a higher LRV than the other two reflection types at the lowest reflectivity. Moreover, as the inner-wall reflectivity grows, the maximum value of fluence rate within UV reactors with highly reflective walls are clearly changed to higher values (see Supplementary Fig. S3 online). It should be noted that increasing the reflectivity of the reactor surface raises the average value of fluence rate and lowers the uniformity of fluence distribution, as found in “Effect of number of UV lamps” by increasing the number of UV lamps, and higher reflectivity yields better performance. So, the influence of a more uniform distribution was diminished once again as a result of a higher average value of the fluence rate.

### Comparison with traditional UV reactor

In this study, some of the dimensions and operating conditions were inspired from the L-shaped UV reactor studied by Sozzi and Taghipour^19^. Considering that the L-shaped reactor is one of the most popular types of UV photoreactors and provides a better performance compared to the U-shaped arrangement^19^, we do a comparison between the proposed UV reactor of this study and the L-shaped one. For this comparison, the best combination of studied parameters, providing the highest level of inactivation, are chosen. Table 3 compares LRV between the proposed and the L-shaped reactor. Note that both the experimental data and our own simulation results have been provided for the conventional L-shaped photoreactor. Based on the comparison of CFD results, the performance of the new reactor is equal or marginally better than the L-shaped when proper choices are made for the new design. Compared to the experimental data, the same conclusion can be drawn considering the uncertainties exist in the experimental data such that two different LRVs are obtained at the same flow rate.

**Table 3:** Comparison of proposed configuration LRVs with experimental and computational data of L-shaped reactor at different volumetric flow rates.

Q [GPM]	Experimental LRV Sozzi and Taghipour^19^	LRV of simulated L-shaped reactor	LRV of proposed system
10	3.4	3.17	3.48
15	(2.73, 3)	2.62	2.66
20	–	2.1	2.16
25	(1.52, 2.1)	1.8	1.82

As mentioned in “Introduction”, the suggested arrangement in this research provides significant benefits over the conventional reactors. Therefore, this study's reactor design is highly promising for the same water disinfection performance as the typical arrangements without their known issues.

## Conclusions

In this paper, UV water disinfection process in a novel configuration was presented and explored using CFD simulations. The reactor's shape consists of two concentric cylinders. The inner cylinder is made of quartz, which has high UV transmittance. The outer cylinder works as a reflector made of highly UV reflective materials. The UV lamps are installed between the two cylinders. Considering that the UV lamps are not in touch with water, this design does not have many of fouling and maintenance issues associated with the traditional reactors. In terms of design parameters, the number of UV lamps, wall reflectivity and its diffuse fraction, and lamp-to-channel distance, were considered to evaluate the reactor performance. The flow field was simulated using the realizable *k*–*ε* turbulence model and the radiation field was obtained using the DO model. The inactivation ratio was obtained using an Eulerian framework. The CFD model was validated with the existing experimental data for a UV reactor working based on reflections from the wall.

Our results suggest that as the number of UV lamps increases, LRV clearly diminishes. It was shown that the two-lamp arrangement is the best among the studied cases. It was also observed that the LRV decrease obtained by lowering the diffuse fraction was more significant than increasing the number of UV lamps. In addition, the mode of reflection from the cylindrical reflector can significantly affect the performance of the reactor. At small diameters of the reflector, reactor’s LRV for the fully diffuse and half-specular modes are considerably higher than that of the full specular mode. Our results showed that the full diffuse mode outperforms the other cases. The new reactor’s performance decreases in all reflection modes when the UV lamps move away from the channel. The fully specular wall reflection contributed notably to the impact of lamp-to-channel distance on LRV. As the lamp-to-channel distance increases from 7.5 to 15 cm, the LRV of fully specular mode decreases about 30%. Moreover, as the lamp-to-channel distance grows, the difference between the LRVs of the fully specular and fully diffuse modes increases from 10 to 47%.

Regardless of the diffuse fraction, increasing the reflectivity of the reflector’s walls creates an increasing trend in LRV. An essential role of high wall reflectivities was observed in determining the impact of the diffuse fraction on reactor performance. It was shown that for smaller wall reflectivities, the mode of reflection changes the microbial performance only slightly.

Finally, it was shown that the proposed reactor can yield the same or slightly better performance compared to the traditional L-shaped UV photoreactors. Therefore, the new design can be an alternative to the traditional ones as it does not have well-known issues such as not being effective for low UVTs of water, quarts sleeve fouling, difficult maintenances, overheating in low flow rates, and exposure to contaminated water. In our future work, we intend to conduct experimental investigations on this proposed reactor and optimize the geometry of this new type of reactors and enhance their microbial performance.

## Supplementary Information

Supplementary Figures.

## Data Availability

The datasets used and/or analysed during the current study available from the corresponding author on reasonable request.

## Acronyms

CFD: Computational fluid dynamics
DO: Discrete ordinates
GCI: Grid Convergence Index
LRV: Log-reduction value
REF: Reduction equivalent fluence (mJ/cm^2^)
RTE: Radiation transport equation
UV: Ultraviolet
UVT: Ultraviolet transmittance

## Roman symbols

$${a}_{\lambda }$$: Absorption coefficient (1/m)
$D$: UV dose (mJ/cm^2^)
${D}_{eff}$: Effective diffusion coefficient (m^2^/s)
$${f}_{d}$$: Diffuse fraction
$I$: UV intensity (mW/cm^2^)
${I}_{b\lambda }$: Black body intensity (W/m^2^)
$${I}_{\lambda }$$: Radiation intensity (W/m^2^)
$k$: Kinetic rate constant (cm^2^/mJ)
$N$: Numbers of active pathogens per unit volume (PFU/mL)
$$n$$: Refractive index
${N}_{0}$: Initial numbers of active pathogens per unit volume (PFU/mL)
$$p$$: Pressure (Pa)
$Q$: Volumetric flow rate (GPM)
$${q}_{in}$$: Total energy (W/m^2^)
${R}^{*}$: Microorganisms’ survival ratio
$$R$$: Wall reflectivity
$$\overrightarrow{r}$$: Position vector
$$\overrightarrow{s}$$: Direction vector
$\overrightarrow{s^{\prime}}$: Scattering direction vector
$${U}_{j}$$: Velocity component (m/s)
$${T}_{w}$$: Wall temperature (K)

## Greek symbols

$${\varepsilon }_{w}$$: Internal emissivity
$$\Phi$$: Phase function
$$\lambda$$: Wavelength
$$\nu$$: Kinematic viscosity (m^2^/s)
${\nu }_{T}$: Kinematic turbulent viscosity (m^2^/s)
$$\rho$$: Density (kg/m^3^)
$\sigma$: Stephen–Boltzmann Constant
$${\sigma }_{s}$$: Scattering coefficient
Ω′: Solid angle (sr)
