# Supplementary material for: Development and modeling of a novel type of photoreactors with exterior ultraviolet (UV) reflector for water treatment applications
Source: Sci Rep. 2023 May 11;13:7696. doi: 10.1038/s41598-023-34799-0 (PMC10175273; doi:10.1038/s41598-023-34799-0)
Supplement: Supplementary file 1 — Supplementary Figures. [file 41598_2023_34799_MOESM1_ESM.docx]

|  |
| --- |
| (a) |
|  |
| (b) |

**Fig. S1.** Distribution of fluence rate at the mid- plane of reactor for (a) horizontal line, (b) vertical line

| 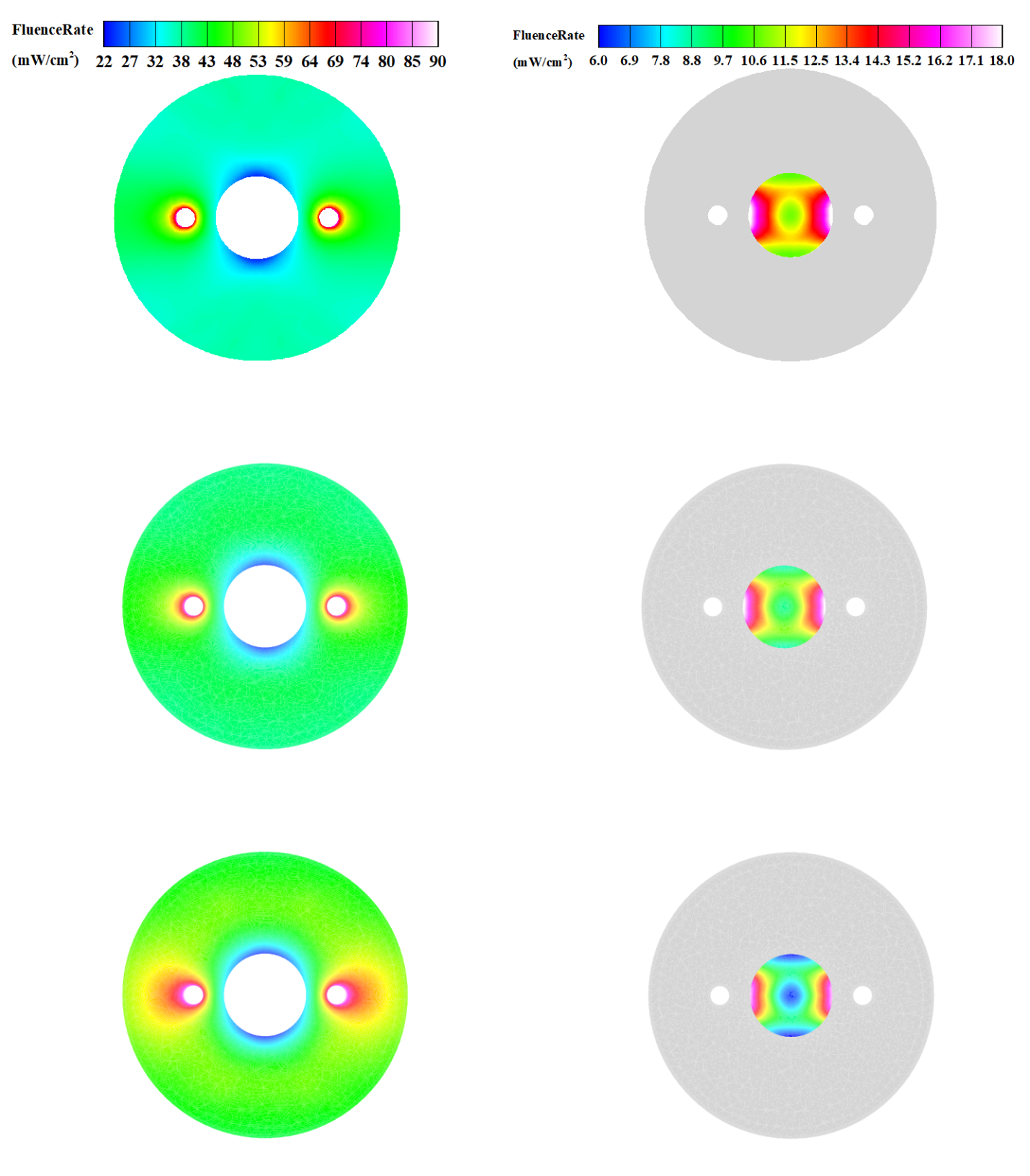 | 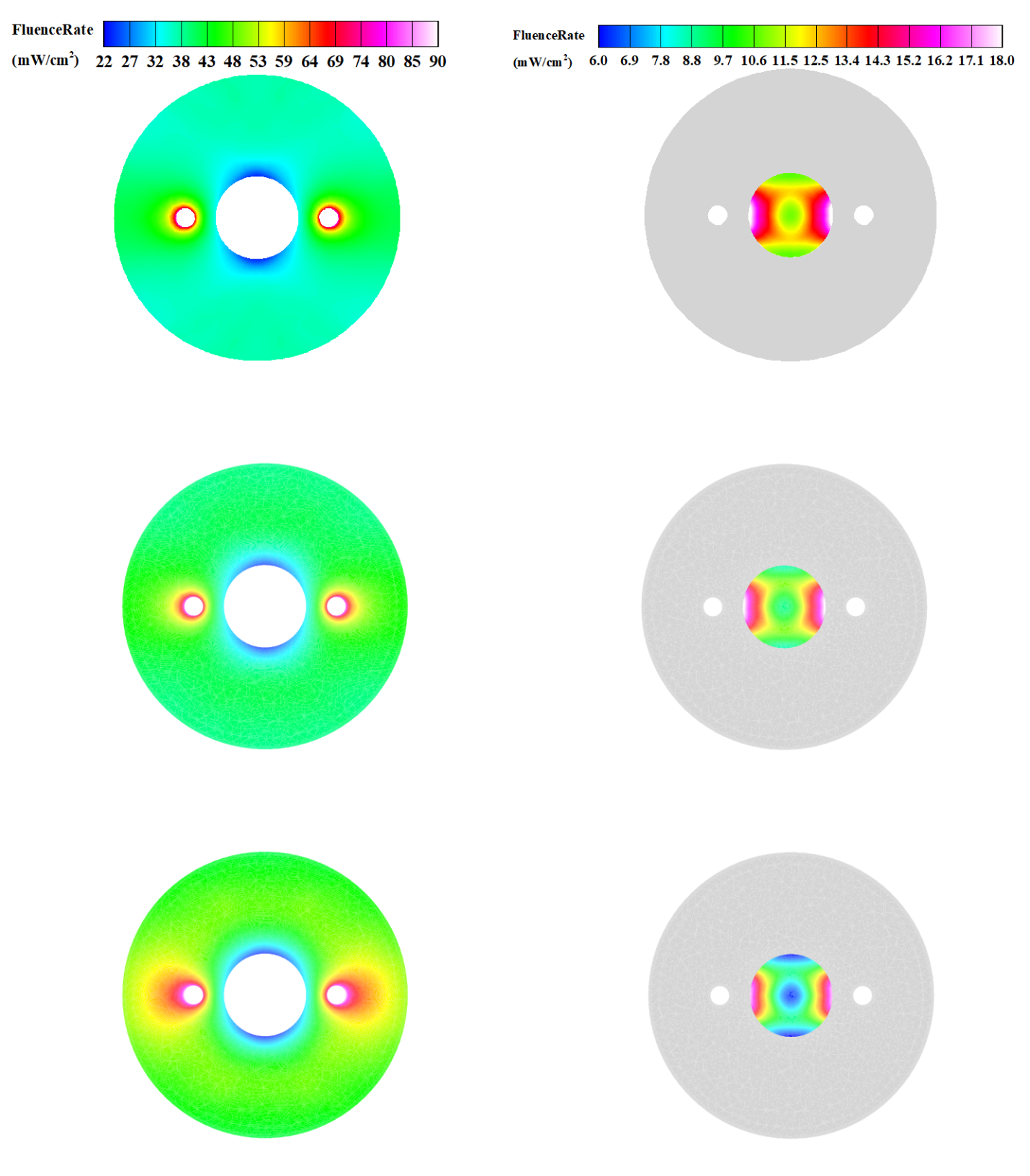 |
| --- | --- |
| (a) | |
| 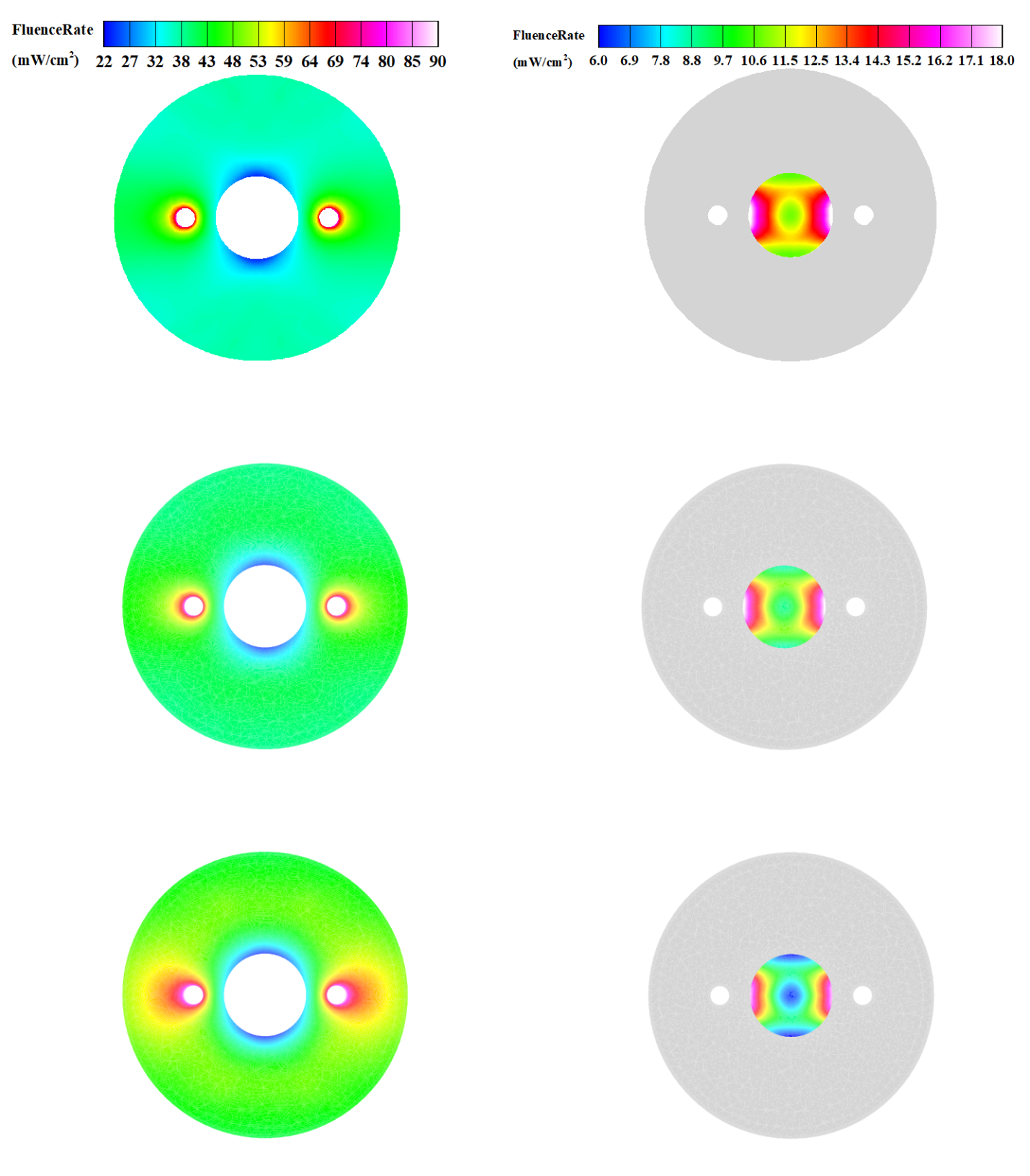 | 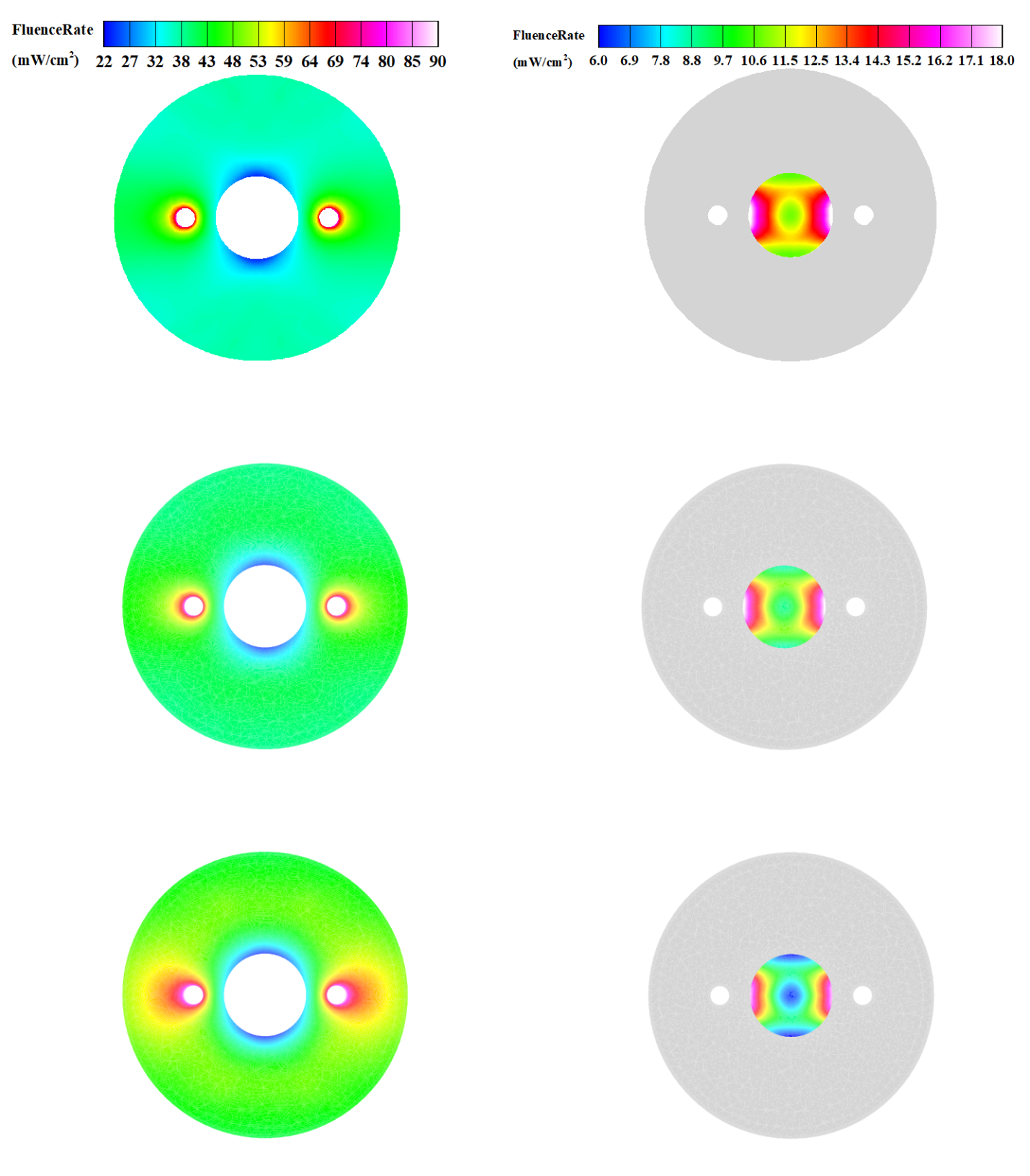 |
| (b) | |
| 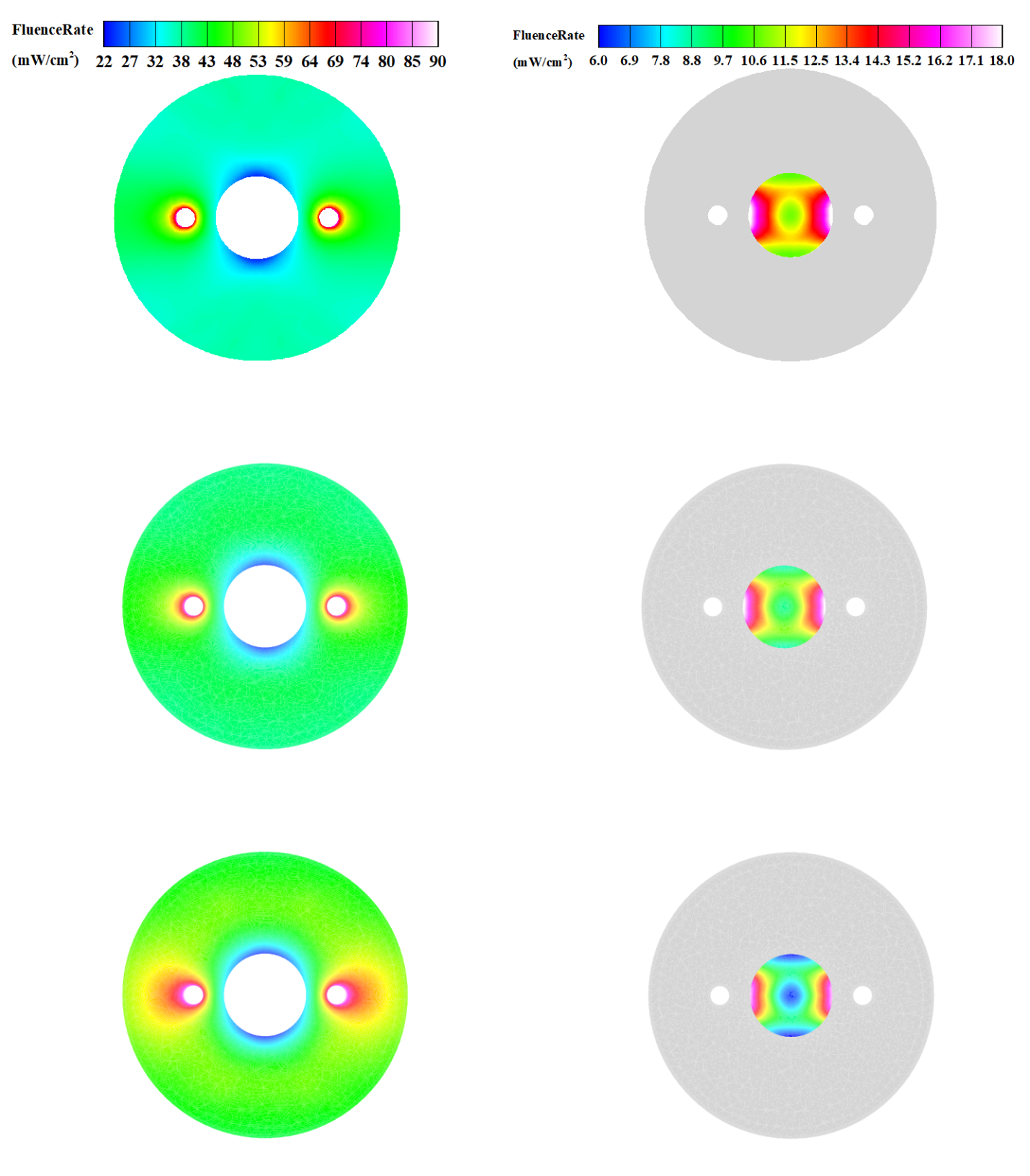 | 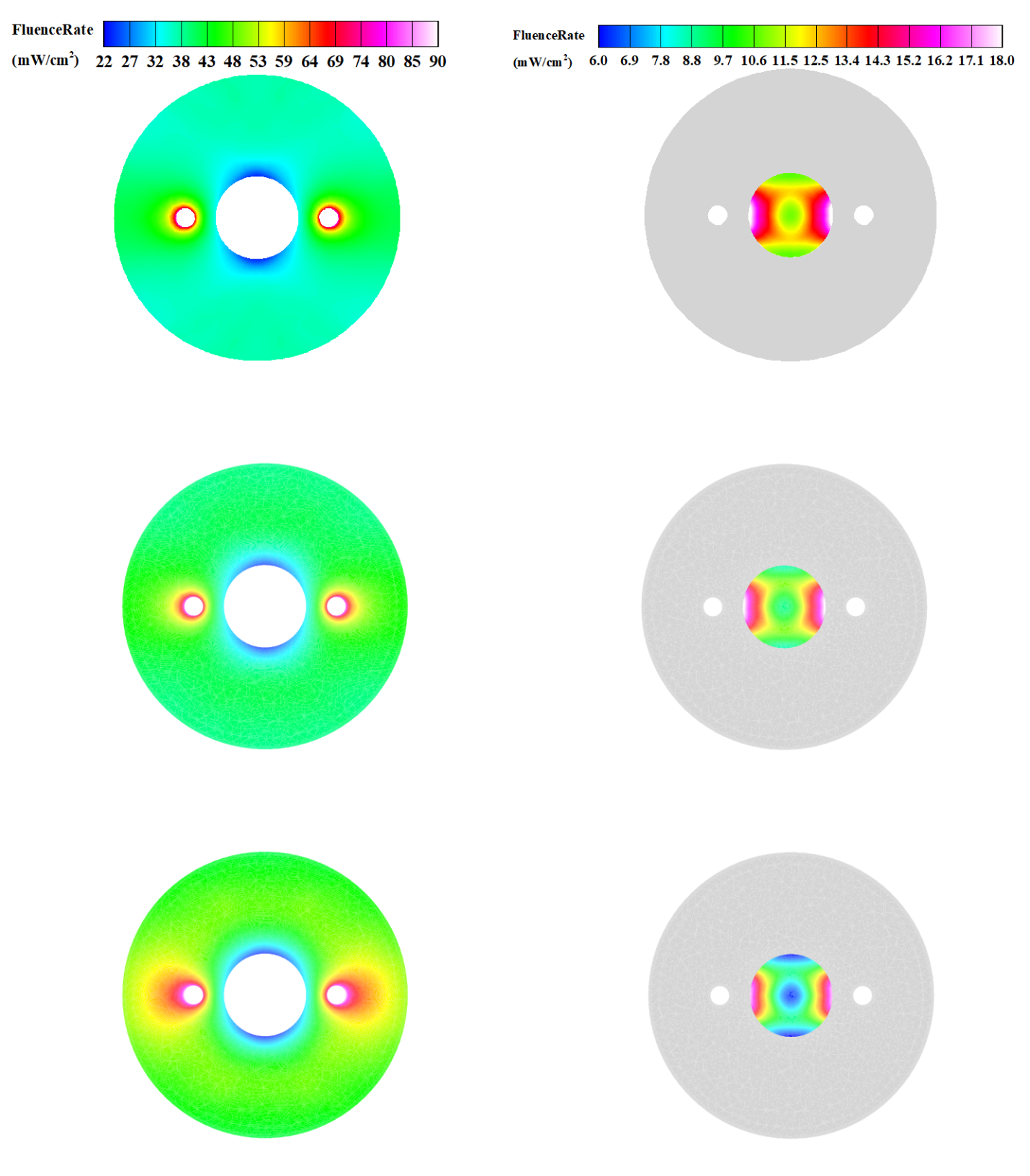 |
| (c) | |

**Fig. S2.** Distribution of fluence rate at the mid- plane of reactor for (a) $f_{d}=1$, (b) $f_{d}=0.5$, and (c) $f_{d}=0$

| 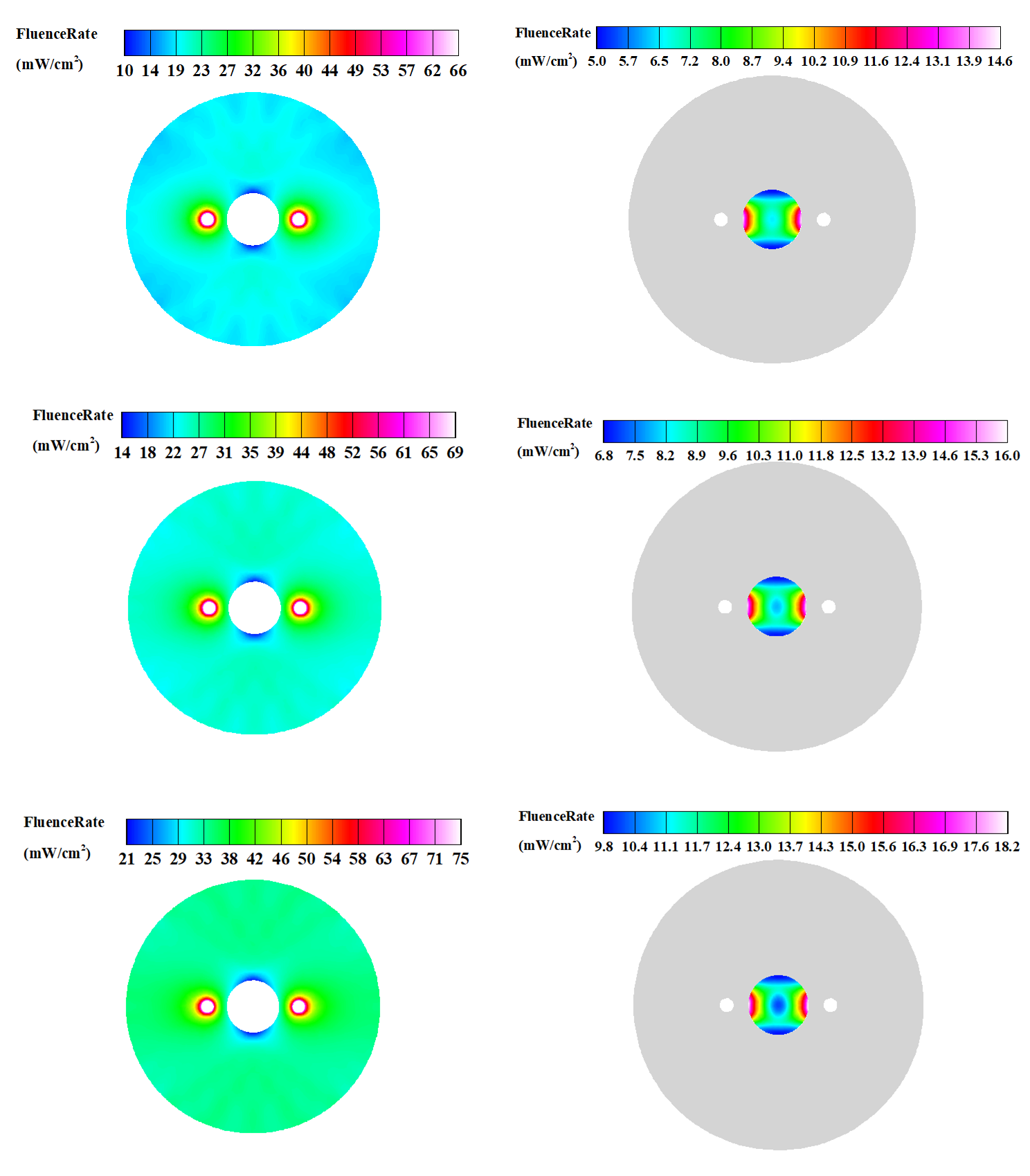 | 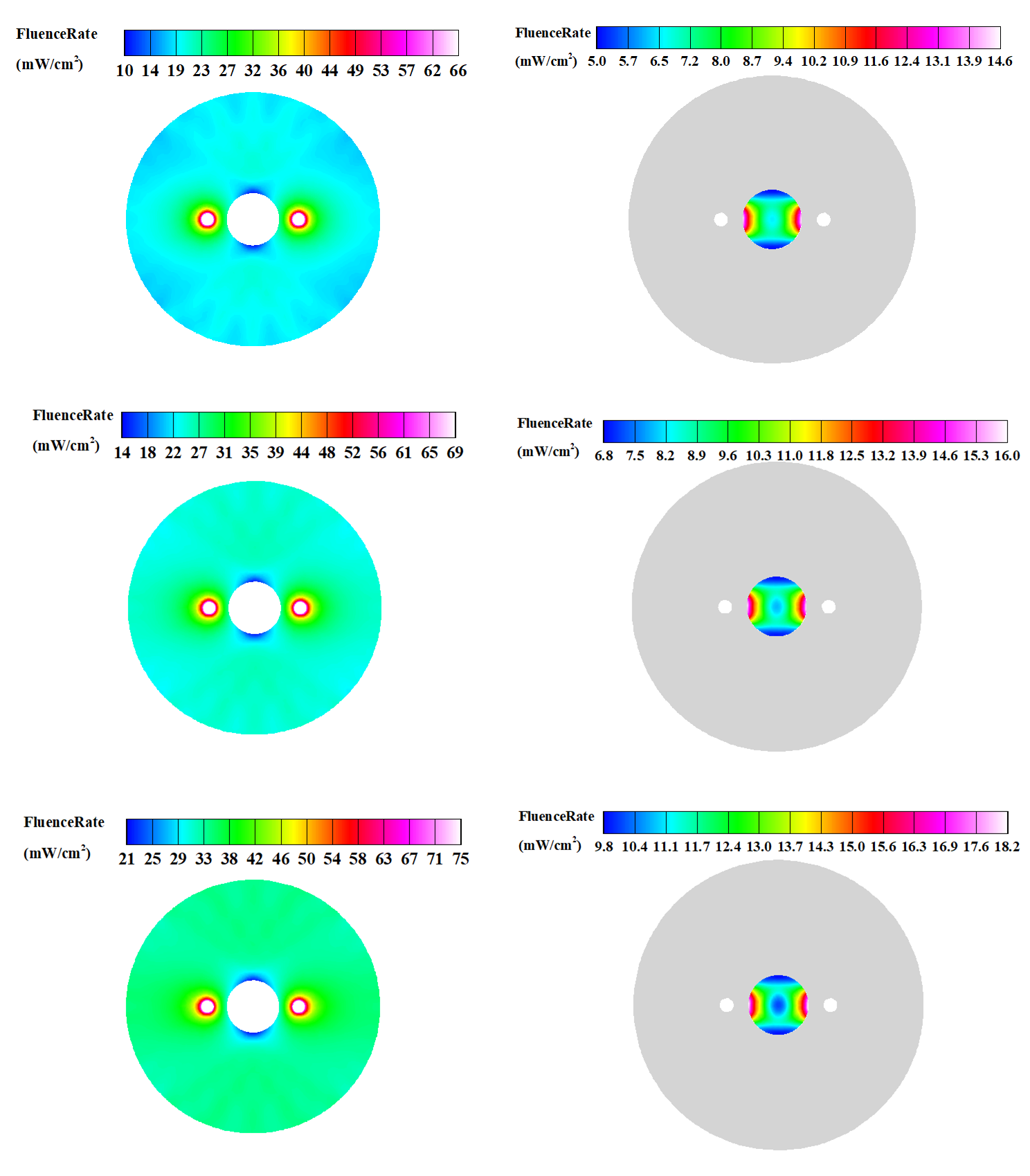 |
| --- | --- |
| (a) | |
| 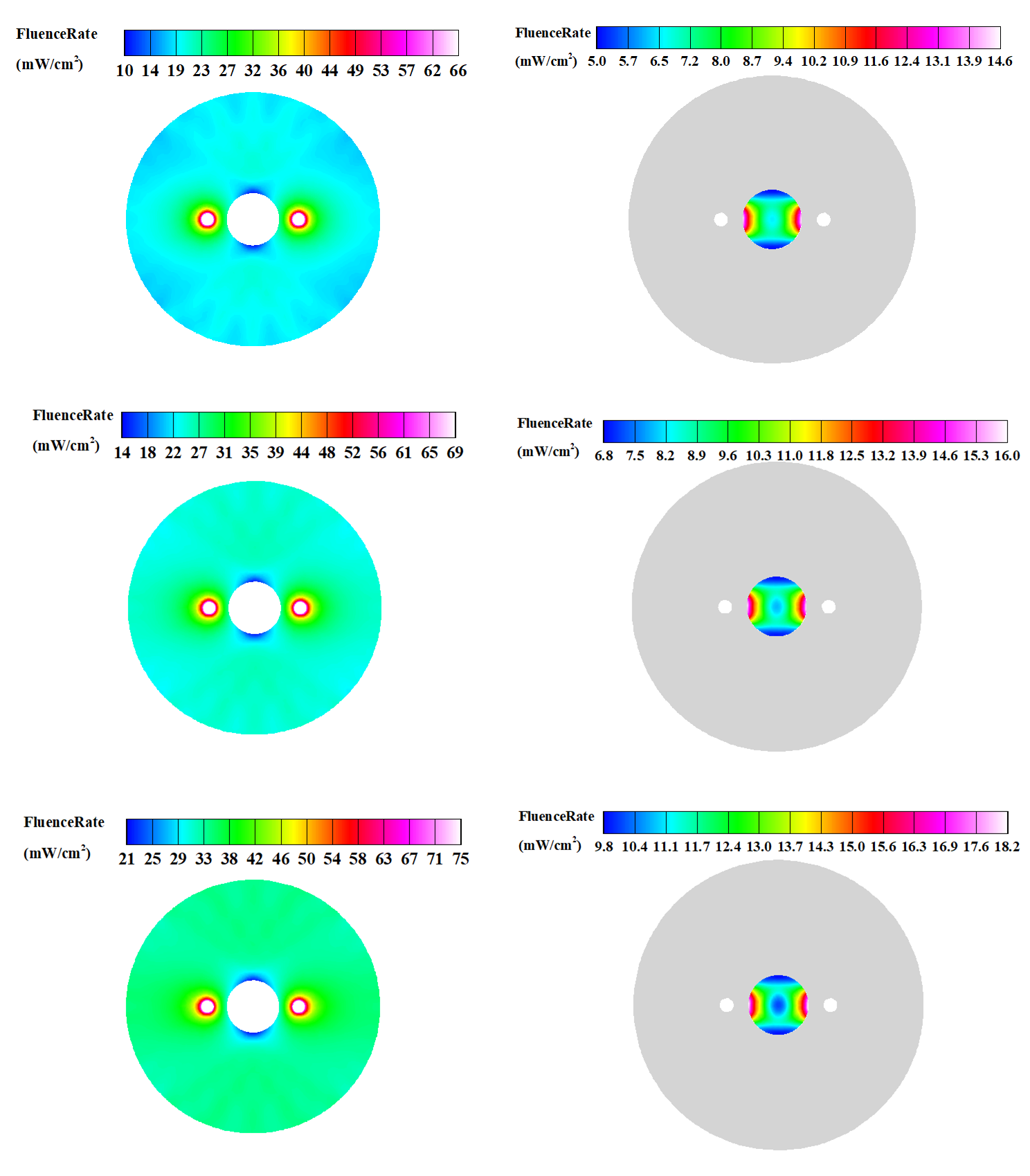 | 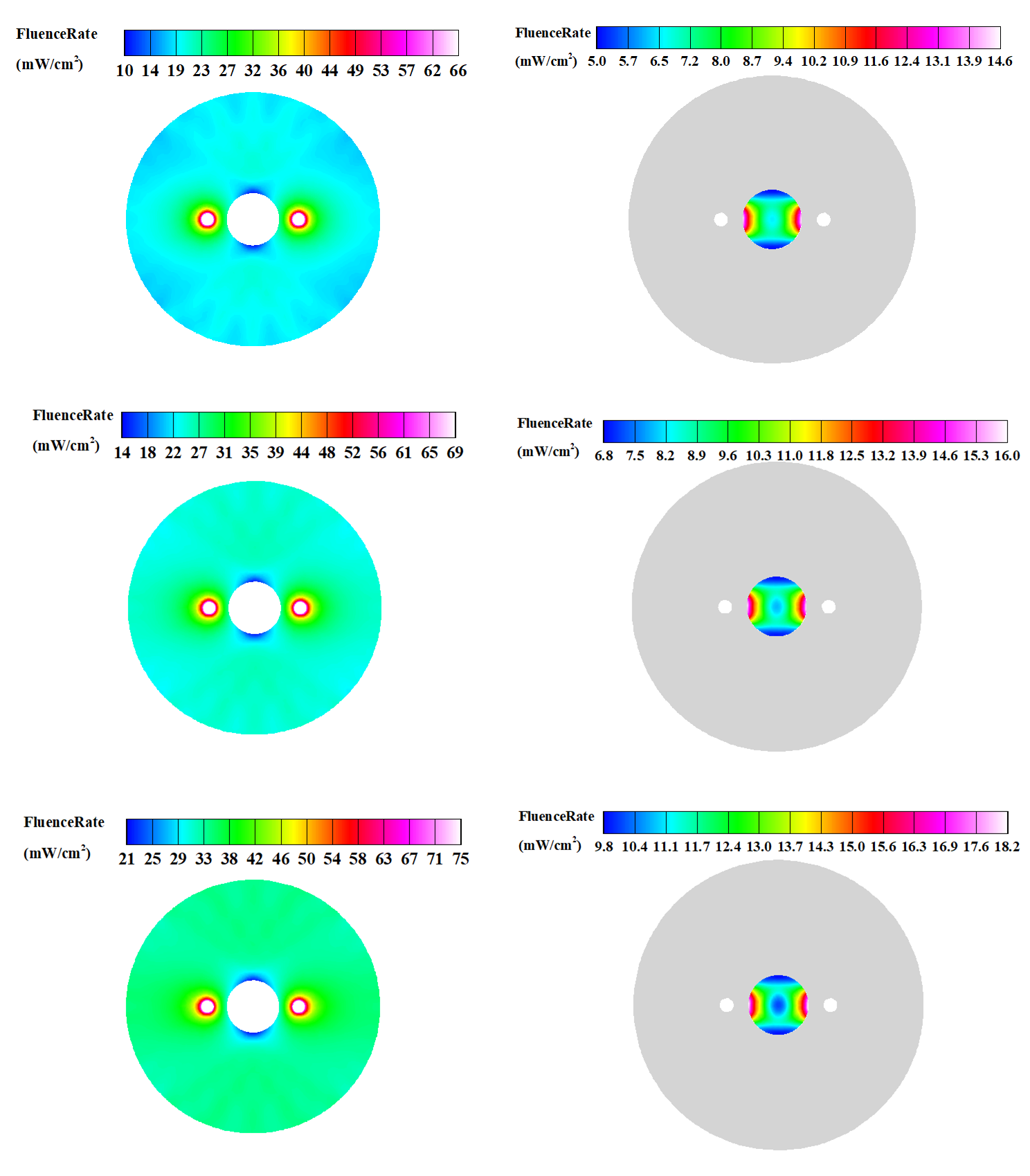 |
| (b) | |
| 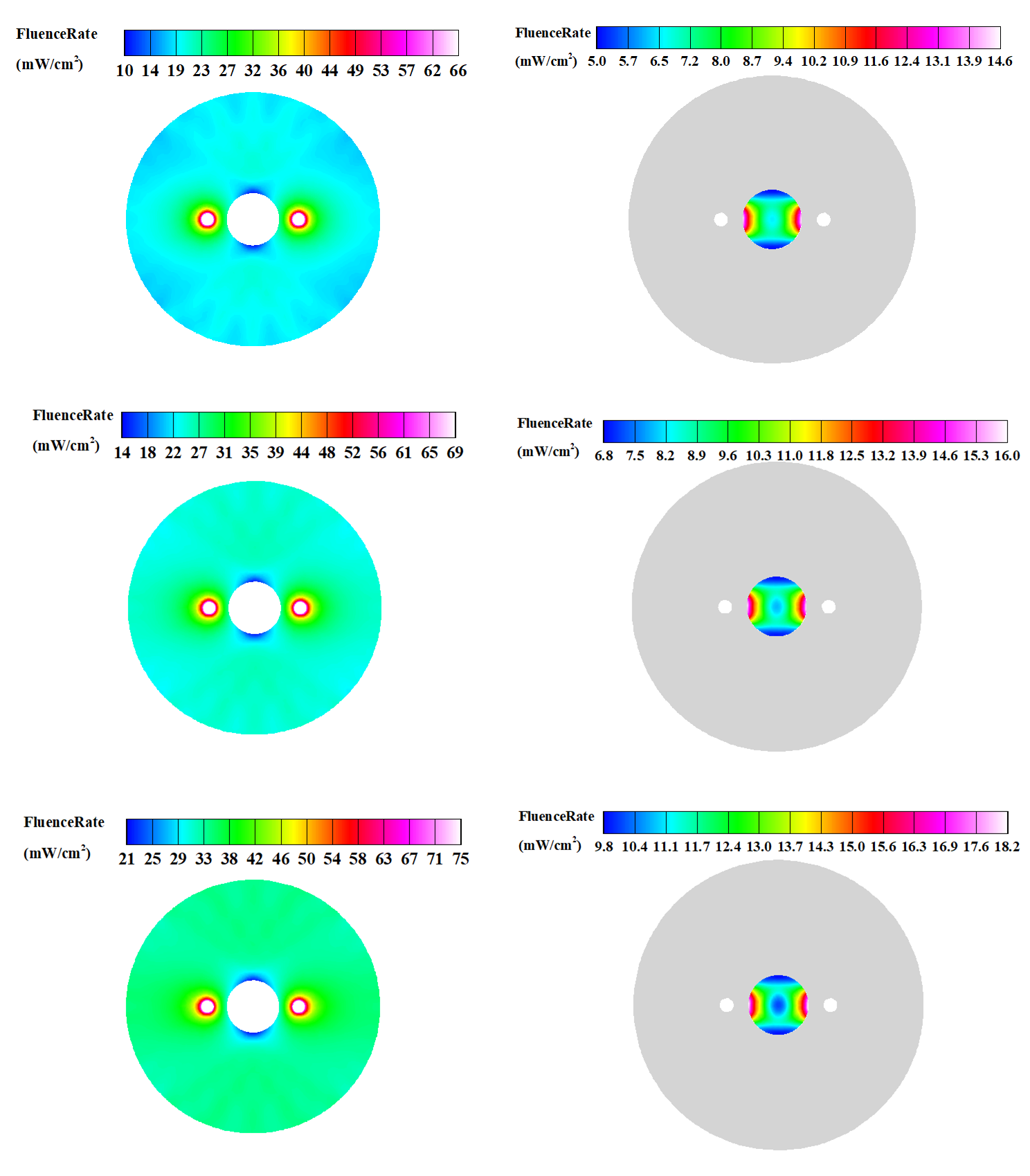 | 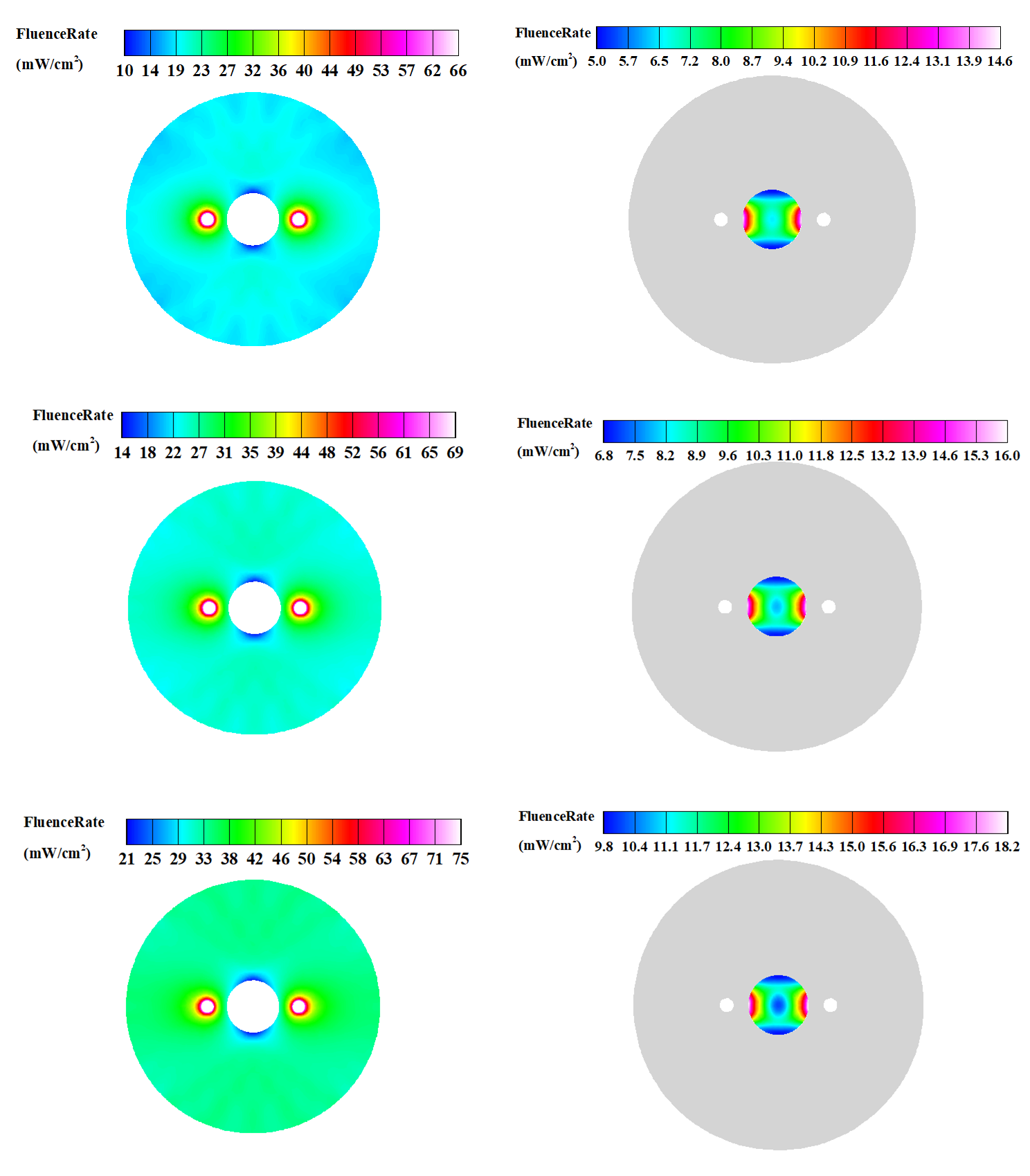 |
| (c) | |

**Fig. S3.** Distribution of fluence rate at the mid-plane of reactor with fully-diffuse UV reflector for (a) $R=0.75$, (b) $R=0.85$, and (c) $R=0.95$.
